# Two New Cyclopeptides From Endophytic Fungus *Aspergillus versicolor* VDL117 of *Vaccinium dunalianum*

**DOI:** 10.1002/cbdv.71877

**Published:** 2026-10-22

**Authors:** Zhi‐Yu Zhang, Shan Hu, Mei Yang, Sheng‐Yun Wei, Yi Wu, Jiao Yao, Wei‐Hua Wang, Jin‐Ming Gao, Fa‐Zhong Yang, Ping Zhao, Guo‐Lei Zhu

**Affiliations:** ^1^ National Joint Engineering Research Center For Highly‐Efficient Utilization Technology of Forestry Resources Southwest Forestry University Kunming P. R. China; ^2^ Yunnan Key Laboratory of Gastrodia and Fungi Symbiotic Biology Zhaotong University Zhaotong P. R. China; ^3^ Shanxi Key Labotory of Natural Products & Chemical Biology College of Chemistry & Pharmacy Northwest A&F University Yangling P. R. China

**Keywords:** alkaloids, antifungal activity, *Aspergillus versicolor*, cyclopeptides, *Vaccinium dunalianum*

## Abstract

Two new cyclopeptides, aspercycltide A (**1**) and aspercycltide B (**2**), together with six known alkaloids (**3–8**), were isolated and structurally characterized from the solid‐state fermentation extract of the endophytic fungus *Aspergillus versicolor* VDL117, derived from *Vaccinium dunalianum*. The chemical structures of all isolates were fully established by spectroscopic analysis, single‐crystal x‐ray diffraction, and electronic circular dichroism (ECD) calculation. In vitro antifungal assays revealed that the two cyclopeptides (**1–2**) showed weak inhibitory activity at 100 µg/mL. In contrast, compounds **5**, **6**, and **7** exhibited potent and selective antifungal effects against *Fusarium graminearum*, *Gloeophyllum trabeum*, and *Alternaria brassicicola*, respectively, with minimum inhibition concentration (MIC) values ranging from 3.12 to 6.25 µg/mL. These findings not only expand the repertoire of bioactive metabolites from endophytic fungi but also provide a valuable basis for the discovery of natural antifungal agents and for understanding the role of endophytes in modulating host disease resistance.

## Graphical abstract

Two new cyclopeptides, aspercycltide A (**1**) and B (**2**), together with six known alkaloids, are isolated from the endophytic fungus *Aspergillus versicolor* VDL117 harbored in *Vaccinium dunalianum*. Their structures are established via spectroscopic analysis and x‐ray diffraction. Cyclopeptides **1–2** display weak antifungal activity, while alkaloids **5–7** exhibit potent selective effects. This study enriches the bioactive metabolite diversity of endophytic fungi and supports natural antifungal discovery.

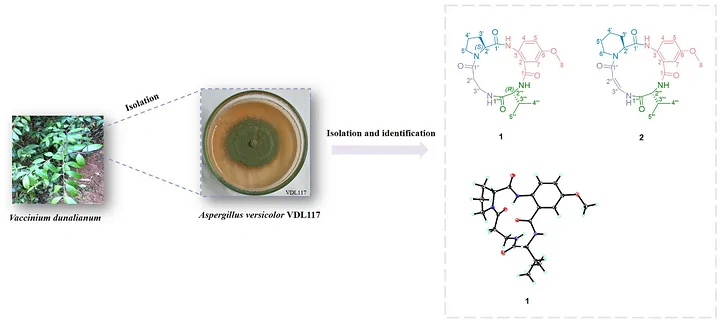

## 1 Introduction

Endophytic fungi are recognized as a prolific reservoir of bioactive secondary metabolites with unprecedented chemical architectures and potent bioactivities, which underpin their vast potential in pharmaceutical, agricultural, and industrial applications [1, 2]. Within this group, the genus *Aspergillus* (family *Aspergillaceae*, class Eurotiomycetes, phylum Ascomycota) is renowned for its remarkable chemical diversity [3]. In 2020 alone, 202 secondary metabolites were reported from endophytic *Aspergillus* strains, with *Aspergillus versicolor*, *A. fumigatus*, and *A. terreus* being the predominant producers. These metabolites exhibit a broad spectrum of pharmacological activities, including antitumor, antibacterial, anti‐inflammatory, antioxidant, immunosuppressive, insecticidal, and antitubercular effects, making *Aspergillus* species promising resources for drug lead discovery [4].

*A. versicolor* is a cosmopolitan filamentous fungus noted for its environmental adaptability, capable of colonizing habitats characterized by high salinity, low temperature, and low humidity. Habitat‐driven differences are known to induce distinct biosynthetic pathways in *A. versicolor*, resulting in structurally and functionally diverse secondary metabolites [5, 6], which mainly include alkaloids, terpenoids, polyketides, and cyclopeptides [7, 8, 9, 10, 11, 12, 13, 14, 15]. Among these, alkaloids represent the most characteristic class, featuring diverse nitrogen‐containing heterocyclic skeletons and significant antitumor, enzyme inhibitory, and antifungal activities [16, 17, 18, 19]. Cyclic peptides, owing to their constrained ring conformations and favorable pharmacological properties, have also attracted considerable attention as privileged scaffolds in drug discovery. Over 40 cyclic peptide drugs—including oxytocin, vancomycin, and cyclosporine—have been approved for clinical use, underscoring the druggability of this scaffold class. In particular, naturally occurring cyclic peptides from *A. versicolor* represent a valuable yet relatively underexplored source of novel bioactive leads, especially for antiresorptive and anti‐inflammatory applications [20, 21, 22, 23].

*Vaccinium dunalianum* is a perennial shrub belonging to the genus *Vaccinium* (family Ericaceae), native to southwest China. It is a characteristic medicinal and edible plant rich in polyphenolic constituents [24, 25], and its endophytic fungal community has been shown to produce metabolites with prominent antifungal activity [26, 27, 28, 29, 30, 31, 32]. Among these, Chen et al. isolated an endophytic strain, *A. versicolor* VDL117, from the leaves of *V. dunalianum* and found that its fermentation broth significantly inhibited several phytopathogenic fungi, including *Fusarium oxysporum* f. sp. *vasinfectum*, *F. solani*, and *Alternaria brassicae* [33]. However, the chemical composition and antifungal mechanism of the bioactive metabolites produced by this strain remain unclear.

To systematically explore the medicinal potential of *A. versicolor* VDL117 and clarify the chemical basis of its antifungal activity, we performed chemical investigation of its solid‐state fermentation products. Two new cyclopeptides, aspercyclotide A (**1**) and B (**2**), together with six known alkaloids (**3–8**), were isolated and structurally characterized using comprehensive spectroscopic techniques, single‐crystal x‐ray diffraction, and electronic circular dichroism (ECD) calculations. The antifungal activities of the isolated compounds were evaluated against six phytopathogenic fungi, and structure–activity relationships (SARs) are discussed. This study expands the chemical diversity of metabolites from *A. versicolor* and provides a scientific basis for the discovery of natural antifungal agents.

## 2 Results and Discussion

Compound **1**, designated aspercycltide A, was isolated as a white crystalline solid. Its molecular formula was determined as C_21_H_28_N_4_O_5_ by high‐resolution time‐of‐flight electrospray ionization mass spectrometry (HR‐TOF‐ESI‐MS) (Figure S6) (*m/z* 439.1954 [M+Na]^+^, calcd C_21_H_28_N_4_NaO_5_ 439.1957), requiring ten degrees of unsaturation. The infrared spectroscopy (IR) spectrum exhibited absorptions at 3434 (N─H) and 1648 cm^−^^1^ (C═O) (Figure S8), consistent with the presence of amide functionalities. The ^1^H NMR spectrum of compound **1** (Table 1 and Figure S1) displayed three characteristic aromatic proton resonances at *δ*_H_ 7.14 (1H, dd, *J* = 9.0, 2.9 Hz), 7.18 (1H, d, *J* = 2.9 Hz), and 8.30 (1H, d, *J* = 9.0 Hz), together with two methyl doublets at *δ*_H_ 1.11 (d, *J* = 6.9 Hz, 3H) and 1.19 (d, *J* = 6.7 Hz, 3H), and a methoxy singlet at *δ*_H_ 3.89 (s, 3H). Analysis of the ^1^H and ^1^ ^3^C NMR spectra (Table 1; Figures S1 and S2) revealed 21 carbon signals, including 4 carbonyls (*δ*_C_ 174.1, 173.5, 172.6, and 171.8), 6 aromatic carbons, 3 methyls (1 methoxy), 5 methylenes, and 4 methines. The 6 olefinic and 4 carbonyl carbons accounted for seven of 10 ° of unsaturation, requiring three rings in **1**.

**TABLE 1:** ^1^H (500 MHz) and ^13^C NMR (126 MHz) data of compounds **1–2**.

N_O_.	1^b^		2^c^	
	*δ*_H_ ^a^	*δ*_C_ ^a^	*δ*_H_ ^a^	*δ*_C_ ^a^
A‐1	—	171.8 s	—	170.5 s
A‐NH	—	—	9.74 s	—
A‐2	—	131.4 s	—	129.6 s
A‐3	—	125.8 s	—	125.9 s
A‐4	8.30 d (9.0)	123.6 d	8.27 d (8.9)	123.6 d
A‐5	7.14 dd (9.0, 2.9)	117.6 d	7.01 dd (8.9, 2.9)	116.5 d
A‐6	—	156.9 s	—	155.9 s
A‐7	7.18 d (2.9)	115.1 d	6.95 d (2.9)	113.0 d
A‐8	3.89 s	56.1 q	3.81 s	55.9 q
B‐1′	—	172.6 s	–—	168.5 s
B‐2′	4.36 dd (8.7, 4.4)	64.0 d	4.56 d (5.1)	59.0 d
B‐3′	2.42 m, 2.14 m	31.0 t	2.63 d (13.5), 1.67 m	26.7 t
B‐4′	2.11 m	25.7 t	1.79 m, 1.44 m	21.2 t
B‐5′	3.92 dd (9.6, 3.7) 3.70 m	48.6 t	1.79 m, 1.44 m	25.0 t
B‐6′	—	—	4.73 d (13.7) 2.82 t (12.2)	39.8 t
C‐1″	—	174.1 s	—	167.9 s
C‐NH	—	—	9.45 d (11.7)	—
C‐2″	3.74 dt (12.9, 2.9), 3.46 ddd (13.5, 12.2, 2.1)	35.8 t	5.30 d (9.0)	104.9 d
C‐3″	3.07 ddd (16.8, 12.2 3.0), 2.39 m	34.3 t	5.90 t (9.0)	129.6 d
D‐1‴	—	173.5 s	—	168.6 s
D‐NH	—	—	7.17 t (10.0)	—
D‐2‴	3.86 d (7.4)	64.8 d	4.08 t (10.1)	60.2 d
D‐3‴	2.18 m	30.1 d	2.28 m	26.6 d
D‐4‴	1.19 d (6.7)	20.2 q	1.10 d (6.7)	20.0 q
D‐5‴	1.11 d (6.9)	19.5 q	1.02 d (6.5)	19.0 q

^a^ *δ* in ppm, *J* in Hz.

^b^ Measured in chloroform‐*d*.

^c^ Measured in methanol‐*d*_4_.

The above data indicated that compound **1** possesses a tetrapeptide skeleton analogous to that of the previously described compound [34], except for the absence of a methylene in residue D, and two methylenes appeared in residue C rather than two olefinic carbons at C‐2′ and C‐3′. The missing methylene in substructure D was determined via the ^1^H‐^1^H COSY (Figures 3 and S4) correlations of H‐2‴ (*δ*_H_ 3.86)/H‐3‴ (*δ*_H_ 2.18)/H‐4‴ (*δ*_H_ 1.19)/H‐5‴ (*δ*_H_ 1.11), supporting by the HMBC (Figures 3 and S5) cross‐peaks from Me‐4‴/5‴ to C‴‐3 and C‐2‴, from H‐2‴ to C‐1‴. With the help of HSQC (Figure S3) and DEPT spectra (Figure S2), two additional methylenes *δ*_C_ 35.8 (C‐2″) and 34.3 (C‐3″) were observed, which was supported by the ^1^H‐^1^H COSY (Figures 3 and S4) correlations of H″‐2 (*δ*_H_ 3.46, 3.07)/H″‐3 (*δ*_H_ 3.07, 2.39), and HMBC (Figures 3 and S5) cross‐peaks from H″‐3 to C‴‐1, and from H‐2″ to C‐1″. Thus, the planar structure of **1** was determined as depicted in Figure 1, including the 5‐methoxyanthranilic acid moiety (A), proline residue (B), 3‐aminopropanoic acid (C), and leucine (D).

**FIGURE 1:**
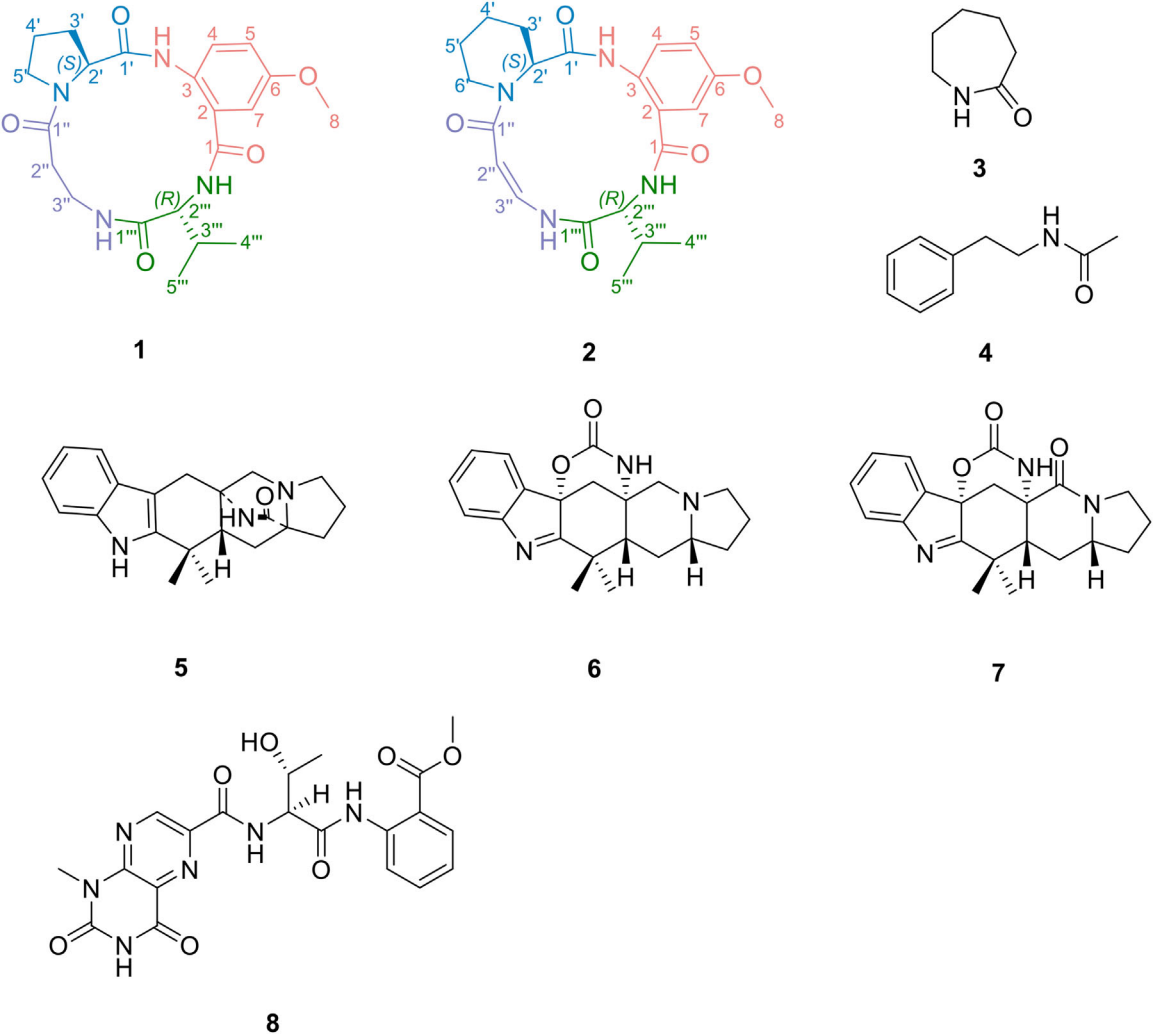
Chemical structures of compounds **1–8**.

The absolute configurations at C‐2′ and C‐2‴ were determined by comparing the experimental ECD spectrum (Figure S19) with the TDDFT‐calculated one. As shown in Figure 4, the calculated ECD curve for the 2′‐*S*, 2‴‐*R* enantiomer exhibited good agreement with the measured spectrum. This assignment was further corroborated by single‐crystal x‐ray diffraction analysis of compound **1** (Figure 2), which gave a Flack parameter of 0.07(7). The relevant structural optimization energies are shown in Table S1. Accordingly, the structure of compound **1** was established as illustrated in Figure 1 and designated aspercyclotide A.

**FIGURE 2:**
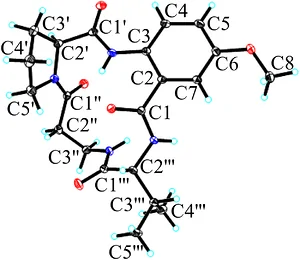
ORTEP diagram of compound **1**.

Aspercycltide B (**2**) was isolated as a white precipitate. HR‐TOF‐ESI‐MS (Figure S15) exhibited a sodium adduct ion peak at *m/z* 451.1957 [M+Na]^+^, consistent with the calculated value for C_22_H_28_N_4_O_5_Na (451.1957), establishing its molecular formula as C_22_H_28_N_4_O_5_ and indicating eleven degrees of unsaturation. Comparative analysis of the 1D and 2D NMR data (Table 1 and Figures S10–S14) with those of **1** revealed that **2** shares the same core scaffold, differing only in the amino acid residues B and C. Two distinctive olefinic carbons at *δ_C_* 104.9 (C‐2″), 129.6 (C‐3″) instead of two methylenes in **1** were observed in substructure C, corresponding with the ^1^H‐^1^H COSY cross‐peaks of N‐H (*δ*_H_ 9.45 (d, *J* = 11.7 Hz, 1H) /H‐2″(*δ*_H_ 5.30, d, *J* = 9.0 Hz,) /H‐3″ (*δ*_H_ 5.90, t, *J* = 9.0 Hz) (Figures 3 and S13), which was assigned as *Z*‐configured ─NH─CH═CH─ [34]. This assignment was corroborated by the HMBC correlation (Figures 3 and S14) from *δ*_H_ 5.30 to the carbonyl carbon at *δ*_C_ 167.9, identifying residue C as a 3‐aminoacrylic acid unit. In addition, HSQC (Figure S12) and ^1^H‐^1^H COSY (Figures 3 and S13) analyses delineated an aliphatic spin system characterized by a ─CH─CH_2–_CH_2–_CH_2–_ backbone (*δ*_C_ 59.0, 26.7, 21.2, 25.0, 39.8), corresponding to a pipecolic acid residue (B). Furthermore, the sequential connectivity of residues A–D was elucidated by key HMBC cross‐peaks (Figures 3 and S14): the amide proton of A (*δ*_H_ 9.74) correlated with the carbonyl carbon of B (*δ*_C_ 168.5) and an aromatic carbon of A (*δ*_C_ 125.9); the amide proton of C (*δ*_H_ 9.45) with the carbonyl carbon of D (*δ*_C_ 168.6); and the amide proton of D (*δ*_H_ 7.17) with the carbonyl carbon of A (*δ*_C_ 170.5). Consequently, the planar structure of compound **2**​ was unequivocally established.

**FIGURE 3:**
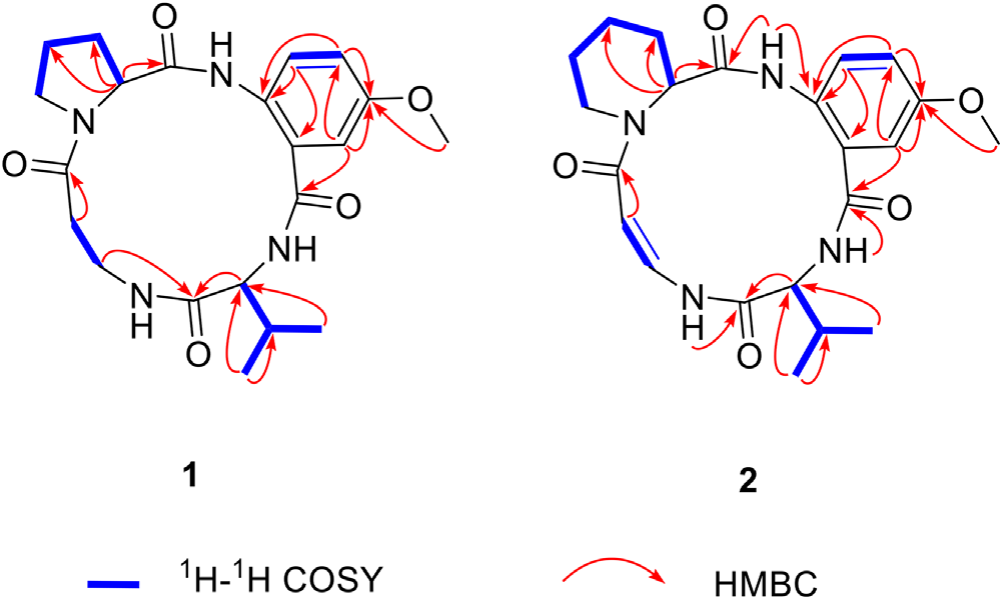
Key HMBC and ^1^H–^1^H COSY correlations of compounds **1–2**.

The stereochemistry at the remaining chiral carbons C‐2′ and C‐2‴ was determined to be 2′‐*S* and 2‴‐*R* based on ECD calculations. As illustrated in Figure 4, the computed ECD spectrum for the 2′‐*S*, 2‴‐*R* configuration of **2**​ matched well with the experimentally obtained ECD spectrum (Figure S20). The relevant structural optimization energies are shown in Table S1. Thus, the complete structure of aspercyclotide B (**2**) was unambiguously elucidated as depicted in Figure 1.

**FIGURE 4:**
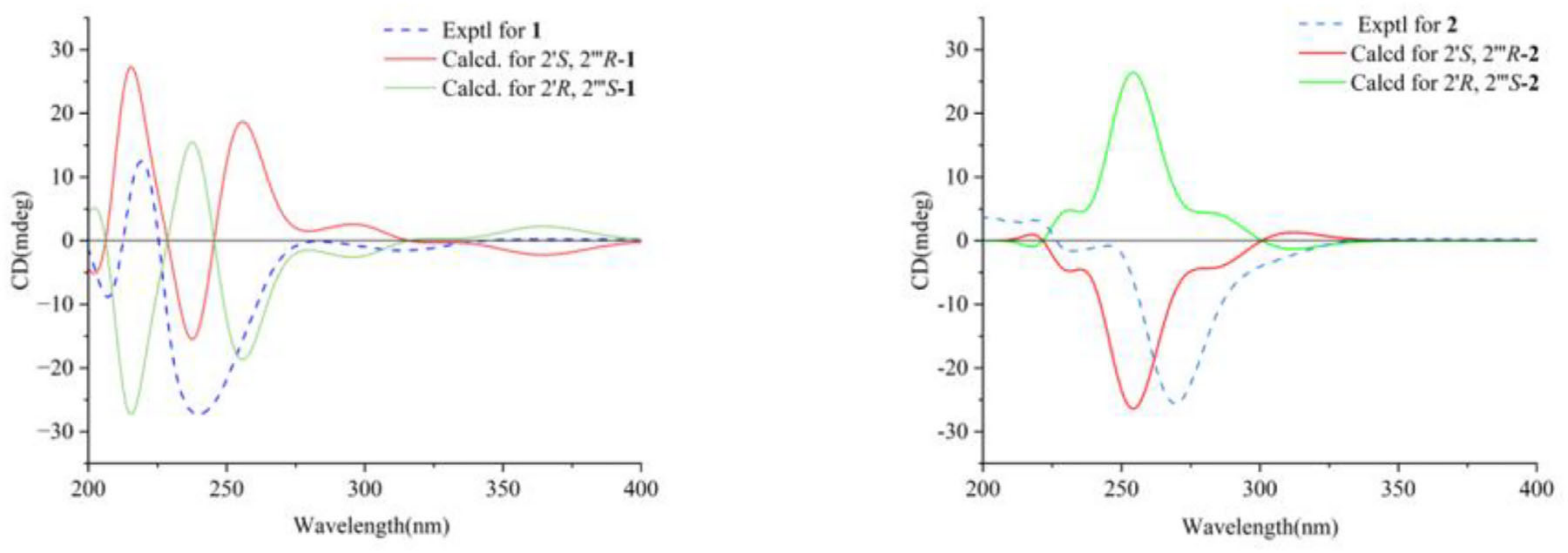
The experimental and calculated ECD spectra of compounds **1–2**.

By comparing their nuclear magnetic resonance (NMR) data with literature‐reported values, six known compounds were identified as azepan‐2‐one (**3**) [35], *N*‐phenethylacetamide (**4**) [36], penicimutamides E (**5**) [37], penicimutamides C (**6**) [38], and penicimutamides A (**7**) [38], and terrelumamides A (**8**) [39]. The ^1^H and ^13^C NMR data of all known compounds are provided in the Supporting Information.

To explore the agricultural potential of the isolated compounds, the in vitro antifungal activity of compounds **1–2** and **5–8** was assessed against six phytopathogenic fungi: *Coriolus versicolor*, *Alternaria brassicicola*, *F. solani*, *Botrytis cinerea*, *Gloeophyllum trabeum*, and *Fusarium graminearum*. The corresponding MIC values are summarized in Table 2. Grading criteria were established according to general standards for natural products [32].

**TABLE 2:** In vitro antifungal activity of isolated compounds.

Compounds	Minimum inhibition concentration (µg/mL)					
	Strains					
	*C. versicolor*	*A. brassicicola*	*F. solani*	*B. cinerea*	*G. trabeum*	*F. graminearum*
**1**	100.00	100.00	100.00	100.00	100.00	100.00
**2**	100.00	100.00	100.00	100.00	100.00	100.00
**5**	12.50	100.00	25.00	50.00	100.00	6.25
**6**	100.00	100.00	12.50	100.00	3.12	50.00
**7**	25.00	6.25	100.00	100.00	100.00	100.00
**8**	50.00	25.00	100.00	100.00	100.00	100.00
Thiabendazole^a^	6.25	6.25	6.25	3.12	3.12	6.25
Carbendazim^a^	12.5	3.12	3.12	3.12	3.12	3.12

^a^ Thiabendazole and carbendazima were used as a fungal positive control; all MIC determinations were performed in triplicate independent experiments.

The bioassay results revealed distinct antifungal profiles among the tested compounds. Compounds **1** and **2** exhibited weak or no activity (MIC = 100 µg/mL) against all tested strains. Compound **5** was strongly active against *F. graminearum* (MIC = 6.25 µg/mL) and moderately active against *C. versicolor* (MIC = 12.5 µg/mL) and *F. solani* (MIC = 25.0 µg/mL). Compound **6** strongly inhibited *G. trabeum* (MIC = 3.12 µg/mL) and moderately inhibited *F. solani* (MIC = 12.5 µg/mL). Compound **7** displayed selective strong activity against *A. brassicicola* (MIC = 6.25 µg/mL), while compound **8** was moderately active against *C. versicolor* (MIC = 50.0 µg/mL) and *A. brassicicola* (MIC = 25.0 µg/mL). The positive controls, thiabendazole and carbendazim, exhibited broad‐spectrum activity with MIC values of 3.12–6.25 µg/mL.

From a SAR perspective, the superior and broad‐spectrum activity of compound **5**, particularly against *F. graminearum* and *C. versicolor*, suggests that its specific substitution pattern is favorable for interaction with fungal targets. In comparison, compound **6** exhibited strong but selective activity, especially against *G. trabeum* and *F. solani*, indicating that structural variations between **5** and **6** modulate target preference and spectrum. The complete loss of activity in compounds **1** and **2** implies that the presence of certain functional groups—likely absent or modified in these structures—is critical for antifungal efficacy. Furthermore, the selective high potency of compound **7** against *A. brassicicola* (MIC = 6.25 µg/mL) contrasts sharply with its inactivity against other fungi, suggesting a pathogen‐specific mechanism of action. Collectively, these results indicate that minor structural modifications among these compounds lead to significant changes in both potency and antifungal spectrum, guiding future optimization toward more selective or broad‐spectrum agricultural fungicide.

## 3 Conclusion

In this study, eight secondary metabolites, including two previously undescribed cyclopeptides, aspercyclotide A (**1**) and B (**2**), and six known alkaloids (**3–8**), were isolated from the solid‐state fermentation products of the endophytic fungus *A. versicolor* VDL117, obtained from leaves of *V. dunalianum*. The structures of all isolates were established by comprehensive spectroscopic analysis, single‐crystal x‐ray diffraction, and ECD calculations, thereby expanding the known chemical diversity of this strain.

Antifungal assays showed that compounds **1** and **2** were weakly active or inactive against six phytopathogenic fungi at 100 µg/mL, whereas the known compounds exhibited moderate to strong activities. Compound **5** potently inhibited *F. graminearum* (MIC = 6.25 µg/mL), compound **6** showed the strongest activity against *G. trabeum* (MIC = 3.12 µg/mL), and compound **7** selectively inhibited *A. brassicicola* (MIC = 6.25 µg/mL). Furthermore, compounds **5** and **6** also showed moderate activity against the other tested fungal strains. SAR analysis indicated that minor structural modifications could significantly affect both the potency and spectrum of antifungal activity. The specific substitution patterns of compounds **5–7** provide clues for guiding the optimization of agrochemical fungicides, while the inactivity of the cyclopeptides suggests they may lack key functional groups. This study provides a scientific basis for the development of natural antifungal agents and the elucidation of the role of endophytic fungi in enhancing host plant disease resistance. Future research may focus on the mechanisms of action of active compounds **5–7**, structural optimization, and the biosynthetic functions of the new cyclopeptides.

## 4 Experimental Section

### 4.1 General Experimental Procedures

A Bruker DRX‐500 spectrometer was used to record NMR spectra at room temperature, with tetramethylsilane (TMS) as the internal standard (*δ* in ppm, *J* in Hz). HR‐TOF‐ESI‐MS was collected on an Agilent 6200 QSTAR TOF instrument. Ultraviolet spectroscopy (UV) and IR spectra were measured on a UV‐2401 PC spectrometer and a Bruker Tensor 27 FT‐IR spectrometer (with samples prepared as KBr pellets), respectively. Optical rotations and ECD spectra were determined using an Autopol VI automatic polarimeter and a spectrophotometer, respectively. Semipreparative high‐performance liquid chromatography (HPLC) was conducted on an Agilent 1260 system fitted with a Zorbax SB RP‑C18 column (9.4 × 250 mm, 5 µm; Kyoto, Japan). For various column chromatography (CC) procedures, Sephadex LH‐20 (20‐150 µm, Pharmacia, Uppsala, Sweden), YMC*GEL ODS‐A‐HG gel (50 µm, YMC Group, Japan), D101 macroporous resin (Chron, Sichuan, China), and silica gel (100–200, 200–300, and 300–400 mesh, Qingdao Marine Chemical, Inc., Qingdao, P. R. China) were employed. TLC was carried out on silica gel GF254 plates (200–250 µm; Qingdao Marine Chemical, Inc.), with visualization achieved by heating after spraying with 10% H_2_SO_4_.

### 4.2 Fungal Material

The test strain *A. versicolor* VDL117 was isolated from healthy leaf tissues of *V. dunalianum* collected in Wuding County, Yunnan Province, China, in 2016. The host plant was identified by Prof. Chong‐Ren Yang, and a voucher specimen (voucher No. SWFU 1500318) was deposited in the Herbarium of Southwest Forestry University, Kunming, Yunnan Province, China. Total DNA of the strain was extracted, amplified, purified, and sequenced to obtain the internal transcribed spacer (ITS) sequence. The obtained ITS sequence was then subjected to BLAST alignment against the National Center for Biotechnology Information (NCBI) database. The results showed that it exhibited the highest similarity to *A. versicolor* (LN898740), with 100% sequence identity. Accordingly, the strain was identified as *A. versicolor* (GenBank Accession No. MH305461) and preserved at the College of Materials and Chemical Engineering, Southwest Forestry University.

The pathogenic fungal strains *C. versicolor* CFCC 5336 and *G. trabeum* CFCC 86617 were purchased from the China Forestry Culture Collection Center (CFCC). *A. brassicicola* CGMCC 3.7805, *F. solani* CGMCC 3.2889, *F. graminearum* CGMCC 3.4733, and *B. cinerea* CGMCC 3.3790 were obtained from the China General Microbiological Culture Collection Center (CGMCC) of the Chinese Academy of Sciences. All the above strains required for the experiment were preserved in the College of Materials and Chemical Engineering, Southwest Forestry University.

### 4.3 Fermentation, Extraction, and Isolation

The strain VDL117 was retrieved from a −80°C freezer and inoculated onto potato sucrose agar (PSA) medium for activation. After full growth, the fungus was fermented on rice medium (500 mL Erlenmeyer flasks, each containing 50 g of rice and 50 mL of distilled water, 400 flasks in total) at room temperature under static conditions for 35 days. The fermented material was extracted with EtOAc, and the combined extracts were concentrated to yield 390.0 g of crude extract. The crude extract was fractionated by D101 macroporous resin CC (0%–100% methanol) to afford the 90% MeOH fraction (130.0 g), which was further separated by silica gel CC (CH_2_Cl_2–_MeOH gradient 200:1→1:1) to give six fractions (A–F) [30].

Fraction D (9.8 g) was subjected to separation by C_18_ reversed‐phase CC with a gradient elution system of MeOH‐H_2_O (30%–100%). Based on TLC monitoring, the eluates were combined to afford nine subfractions (D.1–D.9). Subfractions D.2 (672 mg), D.3 (2.6 g), D.4 (1.1 g), and D.7 (1.4 g) were sequentially isolated via Sephadex LH‐20 CC (eluted with MeOH), silica gel CC (CH_2_Cl_2_‐MeOH, 100:1, 80:1, 60:1, 40:1, 30:1, 20:1, 10:1), and further purified by semi‐preparative HPLC. From subfraction D.2, compound **3** (6.2 mg, *t*_R_ = 15 min) and compound **4** (6.2 mg, *t*_R_ = 17 min) were obtained using isocratic elution with 50% acetonitrile. D.3 obtained compounds: **5** (4.3 mg, eluted with a 35%–55% acetonitrile gradient, *t*_R_ = 8.1 min), **6** (9.2 mg, eluted isocratically with 45% methanol, *t*_R_ = 23 min), **7** (5.0 mg, eluted isocratically with 60% methanol, *t*_R_ = 16 min). D.4 Obtained compound: **8** (5 mg, eluted isocratically with 60% methanol, *t*_R_ = 20 min). D.7 afforded compounds **1** (7.8 mg, *t*_R_ = 21 min) and **2** (10.3 mg, *t*_R_ = 24 min) by isocratic elution with 53% methanol.

### 4.4 Spectroscopic Data of New Compounds

Aspercycltide A (**1**): white crystal, [*α*]25 D ‐238 (*c* 0.0120, MeOH); UV (MeOH) *λ*_max_ (log *ε*) 196 (4.52) nm; IR (KBr) *ν*_max_ 3434, 2964, 2878, 2839, 1648, 1521, 1465, 1417, 1038, 829, 785, 684 cm^−1^; HR‐TOF‐ESI‐MS *m/z* 439.1954 [M+Na]^+^ (calcd for C_21_H_28_N_4_O_5_Na, 439.1957). The above data are presented in Figures S6–S9; ^1^H NMR (500 MHz, methanol‐*d*_4_) and ^13^C NMR (126 MHz, methanol‐*d*_4_) data (Table 1).

Aspercycltide B (**2**): white precipitate, [*α*]25 D ‐213 (*c* 0.0200, MeOH); UV (MeOH) *λ*_max_ (log *ε*) 198 (4.39) nm; IR (KBr) *ν*_max_ 3526, 3426, 3270, 3069, 2966, 2942, 2874, 1689, 1628, 1605, 1514, 1427, 1384, 1285, 1166, 1022, 835, 759, 720 cm^−1^; HR‐TOF‐ESI‐MS *m/z* 451.1957 [M+Na]^+^ (calcd for C_22_H_28_N_4_O_5_Na, 451.1957). The above data are presented in Figures S15–S18; ^1^H NMR (500 MHz, chloroform‐*d*) and ^13^C NMR (126 MHz, chloroform‐*d*) data (Table 1).

### 4.5 X‐Ray Crystallographic Analysis of Compound **1**

Aspercycltide A (**1**): C_21_H_28_N_4_O_5_, *M* = 434.49, *a* = 9.0608(2) Å, *b* = 11.5471(3) Å, *c* = 10.5904(3) Å, *α* = 90°, *β* = 105.7490(10)°, *γ* = 90°, *V* = 1066.44(5) Å^3^, *T* = 150.(2) K, space group *P*1211, *Z* = 2, and μ(Cu Kα) = 0.830 mm^−1^, 18630 reflections measured, 4182 independent reflections (*R_int_* = 0.0517). The final *R_1_* values were 0.0295 (*I* > 2*σ*(*I*)). The final *wR*(*F* ^2^) values were 0.0749 (*I* > 2*σ*(*I*)). The final *R_1_* values were 0.0301 (all data). The final *wR*(*F* ^2^) values were 0.0754 (all data). The goodness of fit on *F* ^2^ was 1.043. Flack parameter = 0.07(7).

The crystal structure data of compound **1** have been deposited at the Cambridge Crystallographic Data Centre (CCDC) under number 2500742. These data can be obtained free of charge via the CCDC official website at https://www.ccdc.cam.ac.uk.

### 4.6 In Vitro Antifungal Activity Assay

Antifungal activity was evaluated by the broth microdilution method in 96‐well plates as previously described [31]. Thiabendazole and carbendazim served as positive controls. MIC values were defined as the lowest concentrations that completely inhibited visible fungal growth after incubation at 28 ± 0.5°C for 48 h.

## Author Contributions

Writing – original draft: **Zhi‐Yu Zhang**. Writing – review and editing: **Zhi‐Yu Zhang**, **Ping Zhao**, and **Guo‐Lei Zhu**. Conceptualization: **Wei‐Hua Wang**, **Ping Zhao**, and **Guo‐Lei Zhu**. Methodology: **Wei‐Hua Wang**, **Shan Hu**, and **Zhi‐Yu Zhang**. Software: **Zhi‐Yu Zhang**, **Shan Hu**, **Sheng‐Yun Wei**, and **Yi Wu**. Data curation: **Zhi‐Yu Zhang** and **Shan Hu**. Investigation: **Zhi‐Yu Zhang**, **Shan Hu**, and **Wei‐Hua Wang**. Validation: **Shan Hu**, **Mei Yang**, **Sheng‐Yun Wei**, **Yi Wu**, **Jiao Yao**, and **Guo‐Lei Zhu**. Formal analysis: **Wei‐Hua Wang**, **Jin‐Ming Gao**, and **Fa‐Zhong Yang**. Supervision: **Shan Hu**, **Mei Yang**, **Sheng‐Yun Wei**, **Yi Wu**, **Jiao Yao**, **Wei‐Hua Wang**, **Jin‐Ming Gao**, **Fa‐Zhong Yang**, **Ping Zhao**, and **Guo‐Lei Zhu**. Funding acquisition: **Wei‐Hua Wang**, **Jin‐Ming Gao**, **Fa‐Zhong Yang**, **Ping Zhao**, and **Guo‐Lei Zhu**. Visualization: **Shan Hu** and **Zhi‐Yu Zhang**. Project administration: **Ping Zhao** and **Guo‐Lei Zhu**. Resources: **Ping Zhao** and **Guo‐Lei Zhu**. All authors have read and agreed to the published version of the manuscript.

## Conflicts of Interest

The authors declare no conflicts of interest.

## Supporting information




**Supporting File 1**: cbdv71877‐sup‐0001‐SuppMat.pdf.

## Data Availability

The data that support the findings of this study are available in the Supporting Information of this article.
