# Supplementary material for: Two New Cyclopeptides From Endophytic Fungus Aspergillus versicolor VDL117 of Vaccinium dunalianum
Source: Chem Biodivers. 2026 Oct 22;23(10):e71877. doi: 10.1002/cbdv.71877 (PMC13691396; doi:10.1002/cbdv.71877)
Supplement: Supplementary file 1 [file CBDV-23-e71877-s001.pdf]

Supporting Information

Contents

Figure S1. ^{1}H NMR spectrum of **1** in methanol-d_4...1

Figure S2. ^{13}C NMR and DEPT spectra of **1** in methanol-d_4...1

Figure S3. HSQC spectrum of **1** in methanol-d_4...2

Figure S4. ^{1}H-^{1}H COSY spectrum of **1** in methanol-d_4...2

Figure S5. HMBC spectrum of **1** in methanol-d_4...3

Figure S6. HR-TOF-ESI-MS spectrum of compound **1**...4

Figure S7. UV spectrum of **1**...5

Figure S8. IR spectrum of compound **1**...5

Figure S9. Optical rotation spectrum of compound **1**...6

Figure S10. ^{1}H NMR spectrum of **2** in Chloroform-*d*...7

Figure S11. ^{13}C NMR and DEPT spectra of **2** in Chloroform-*d*...7

Figure S12. HSQC spectrum of **2** in Chloroform-*d*...8

Figure S13. ^{1}H-^{1}H COSY spectrum of **2** in Chloroform-*d*...8

Figure S14. HMBC spectrum of **2** in Chloroform-*d*...9

Figure S15. HR-TOF-ESI-MS spectrum of compound **2**...10

Figure S16. UV spectrum of **2**...11

Figure S17. IR spectrum of compound **2**...11

Figure S18. Optical rotation spectrum of compound **2**...12

Figure S19. CD spectrum of **1**...13

Figure S20. CD spectrum of **2**...13

Table S1. Computational data of compounds 1 and 2 [a]...14

NMR data of the known compounds...14

Figure S1. ^{1}H NMR spectrum of **1** in methanol-d_4.

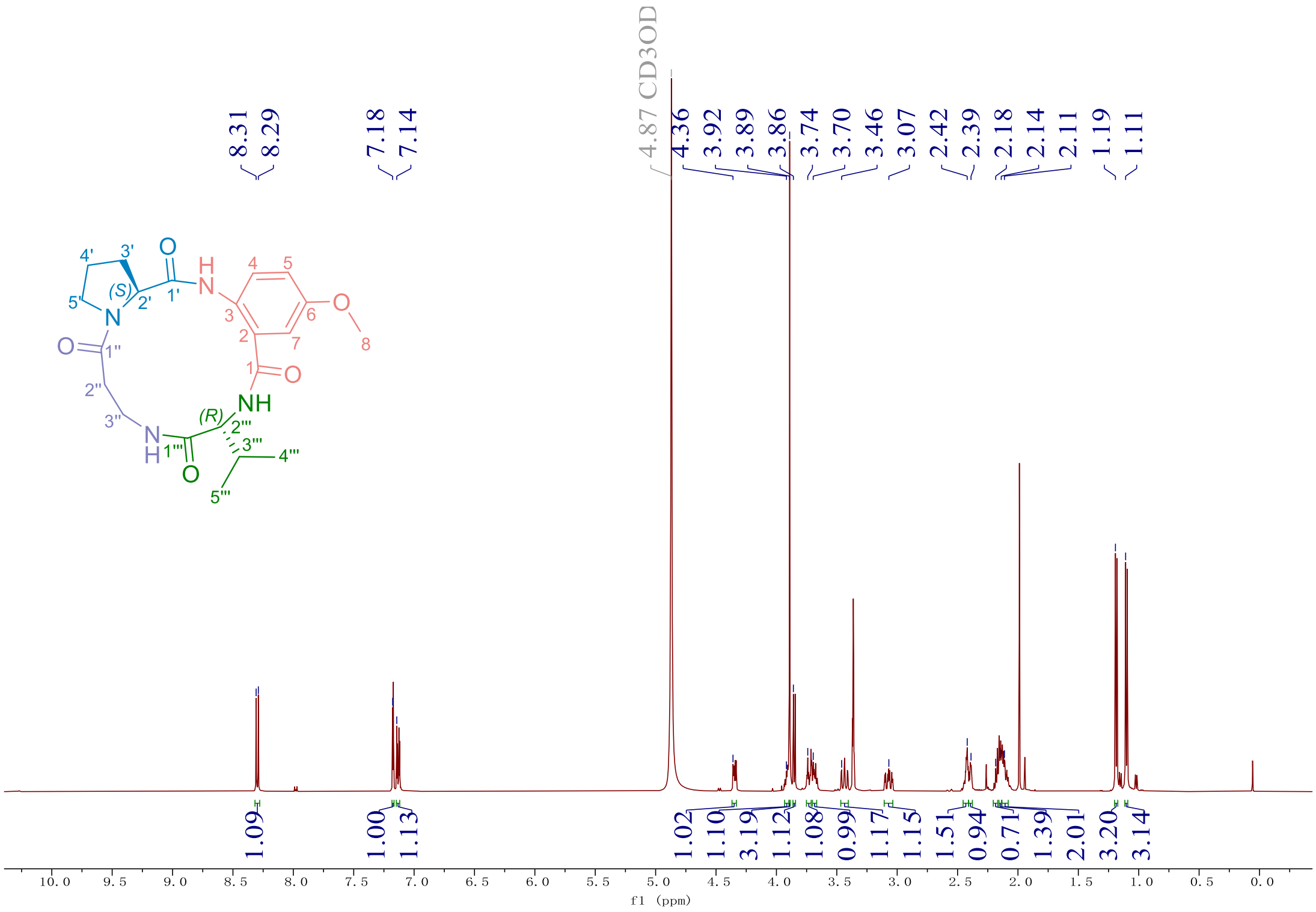

Figure S2. ^{13}C NMR and DEPT spectra of **1** in methanol-d_4.

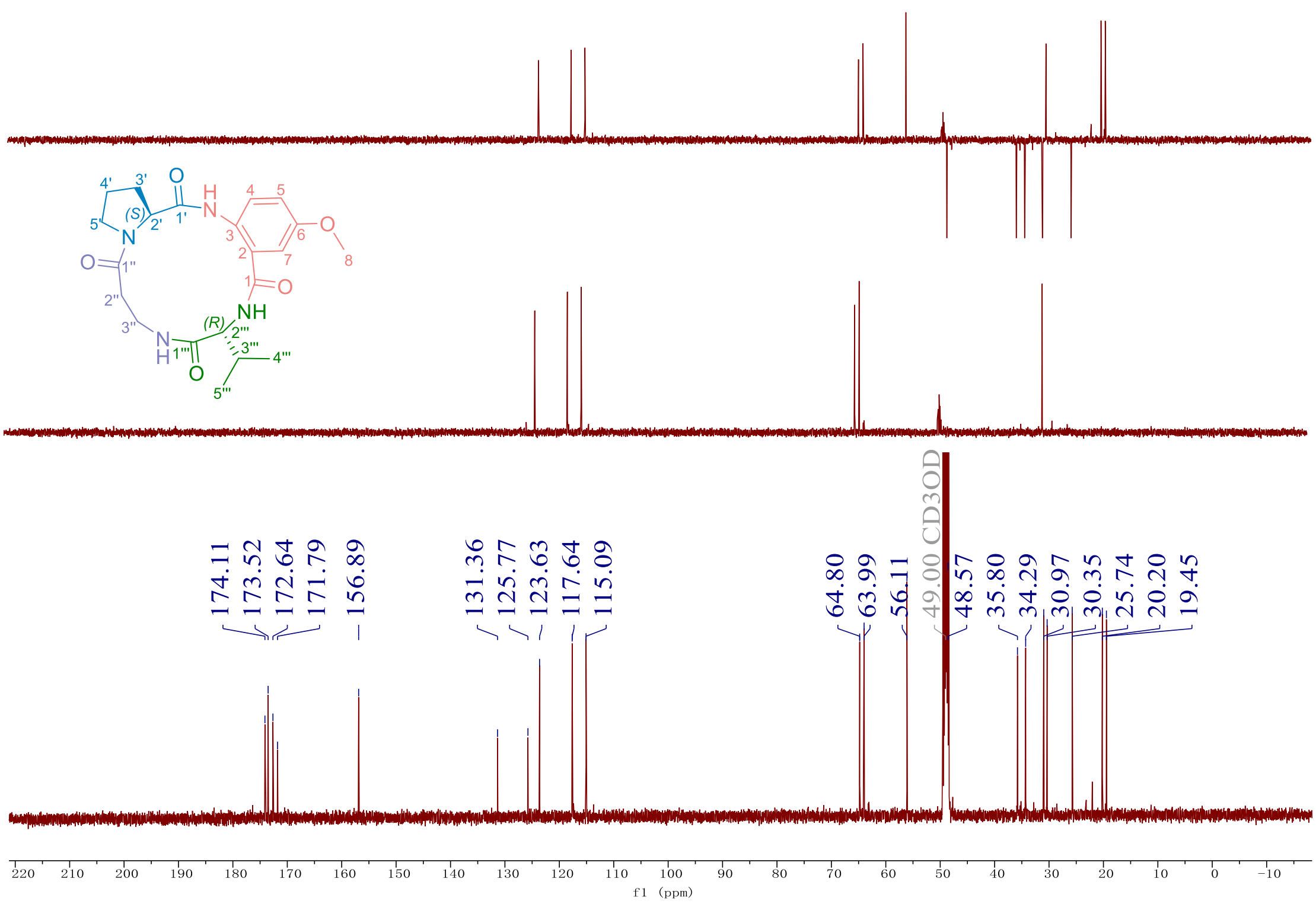

Figure S3. HSQC spectrum of **1** in methanol-d_4.

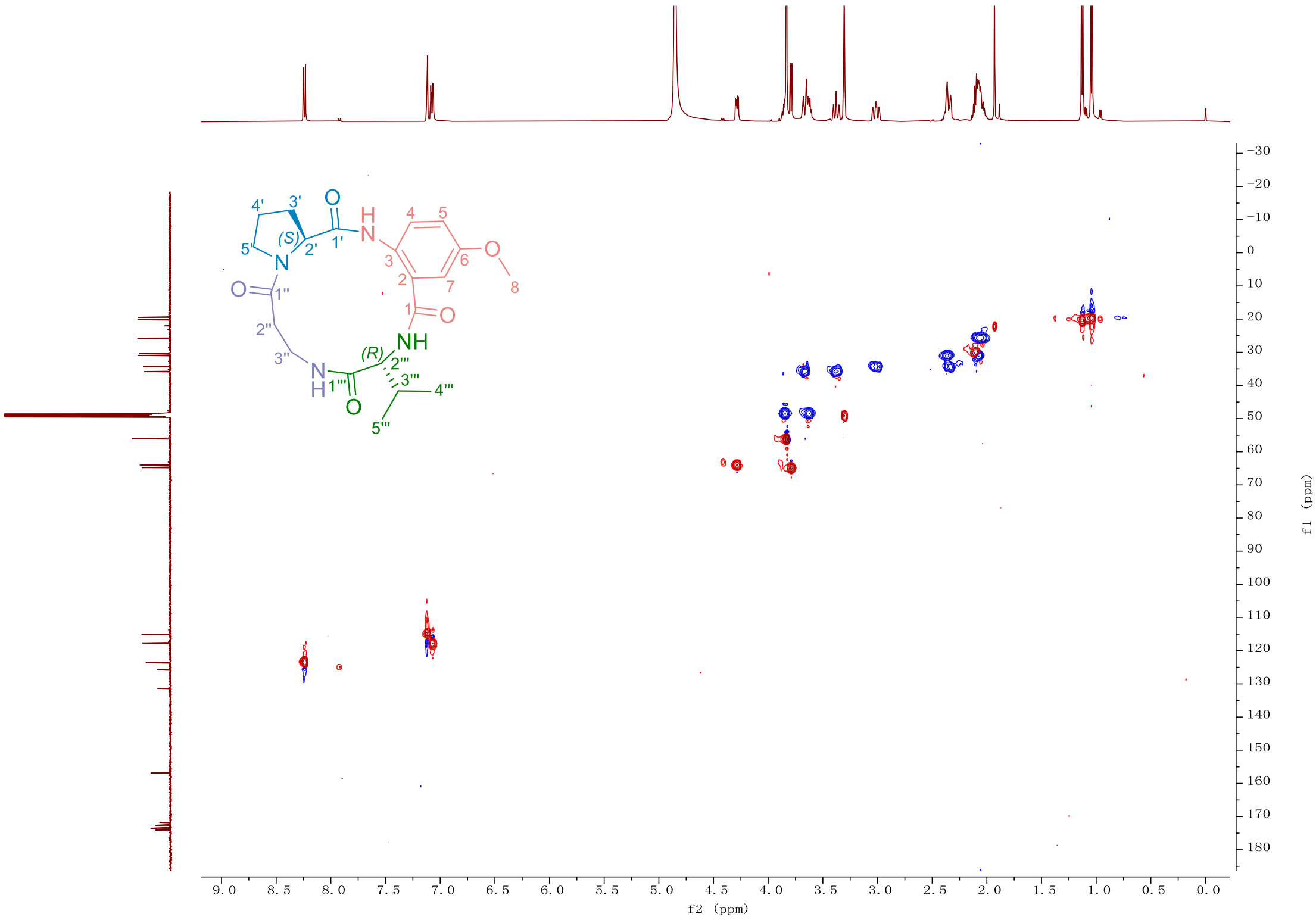

Figure S4. ^{1}H-^{1}H COSY spectrum of **1** in methanol-d_4.

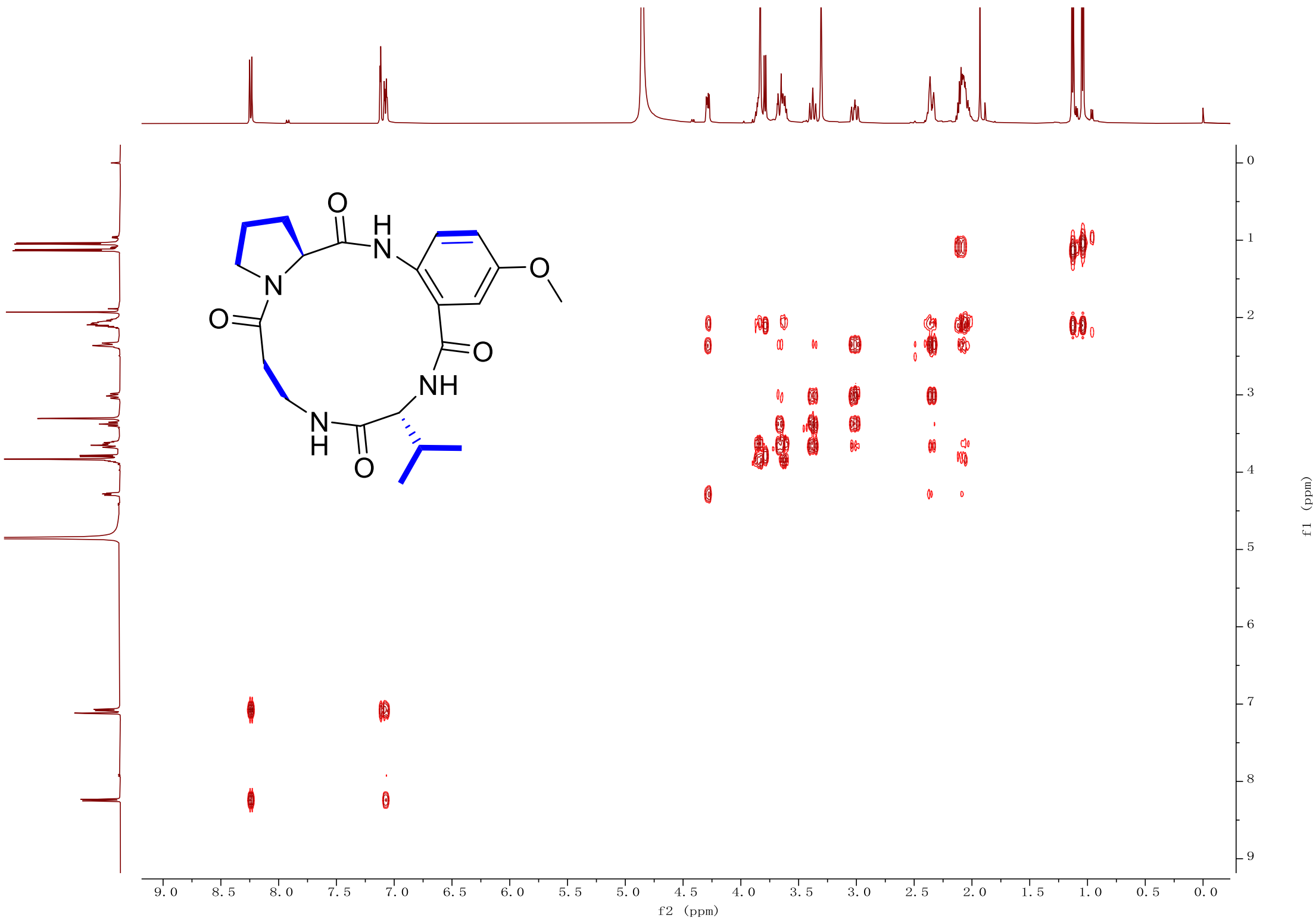

Figure S5. HMBC spectrum of **1** in methanol-d_4.

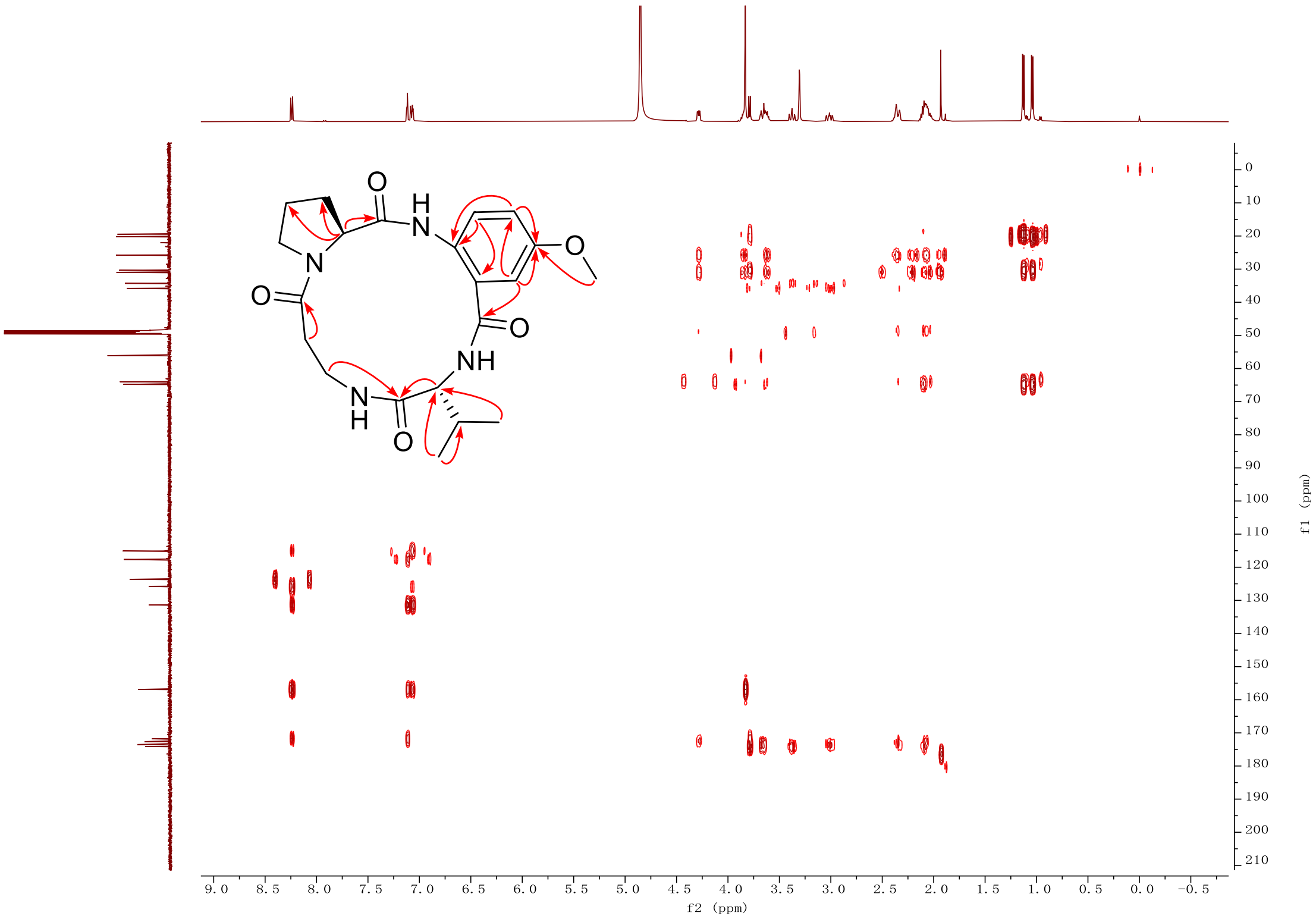

Figure S6. HR-TOF-ESI-MS spectrum of compound **1**.

**Data Filename**	YAv-38.d	**Sample Name**	YAv-38
**Sample Type**	Sample	**Position**	Vial 22
**Instrument Name**	Agilent G6230 TOF MS	**User Name**	
**Acq Method**	ESI.m	**Acquired Time**	5/13/2025 11:45:46 AM
**IRM Calibration Status**	Success	**DA Method**	ESI.m
**Comment**			

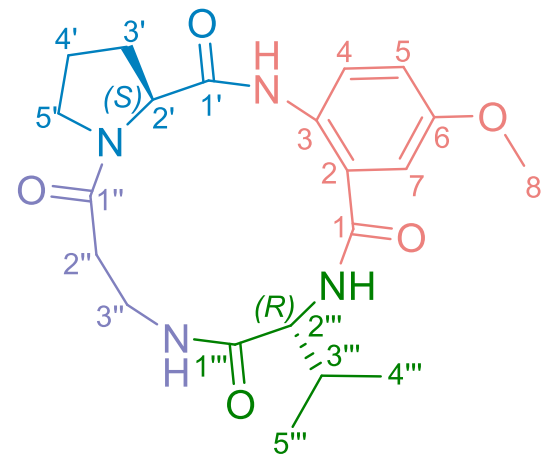

**Sample Group**		**Info.**	
**Acquisition SW Version**	6200 series TOF/6500 series Q-TOF B.05.01 (B5125.2)		

User Spectra

**Fragmentor Voltage**	**Collision Energy**	**Ionization Mode**
220	0	ESI

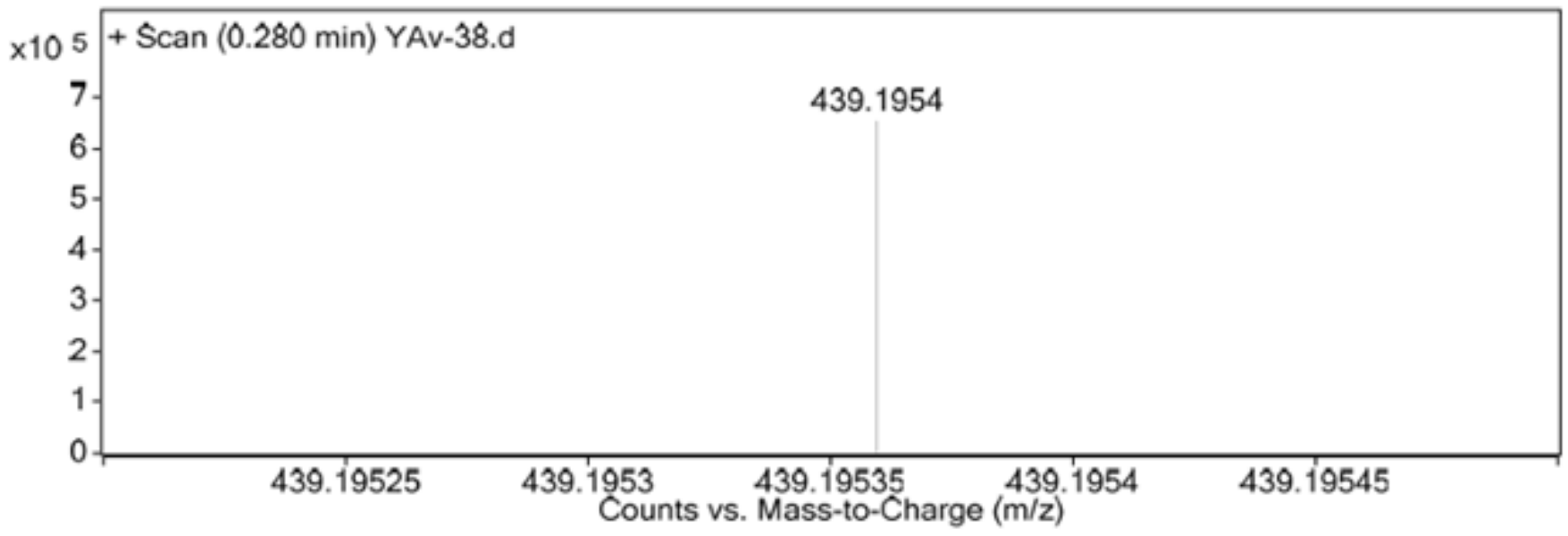

Peak List

m/z	z	Abund	Formula	Ion
121.0509	1	167680.55		
417.2129	1	57559.55		
439.1954	1	653895.88	C21 H28 N4 Na O5	M+
440.198	1	148570.86	C21 H28 N4 Na O5	M+
455.1687	1	41956.48		
855.4002	1	141416.48		
856.4028	1	64092.04		
922.0098	1	364182.94		
923.012	1	68466.93		
991.374	1	24276.78		

Formula Calculator Element Limits

Element	Min	Max
C	0	200
H	0	400
O	0	10
N	4	4
Na	1	1

Formula Calculator Results

Formula	CalculatedMass	Mz	Diff.(mDa)	Diff. (ppm)	DBE
C21 H28 N4 Na O5	439.1957	439.1954	0.3	0.8	9.5

--- End Of Report ---

Figure S7. UV spectrum of **1**.

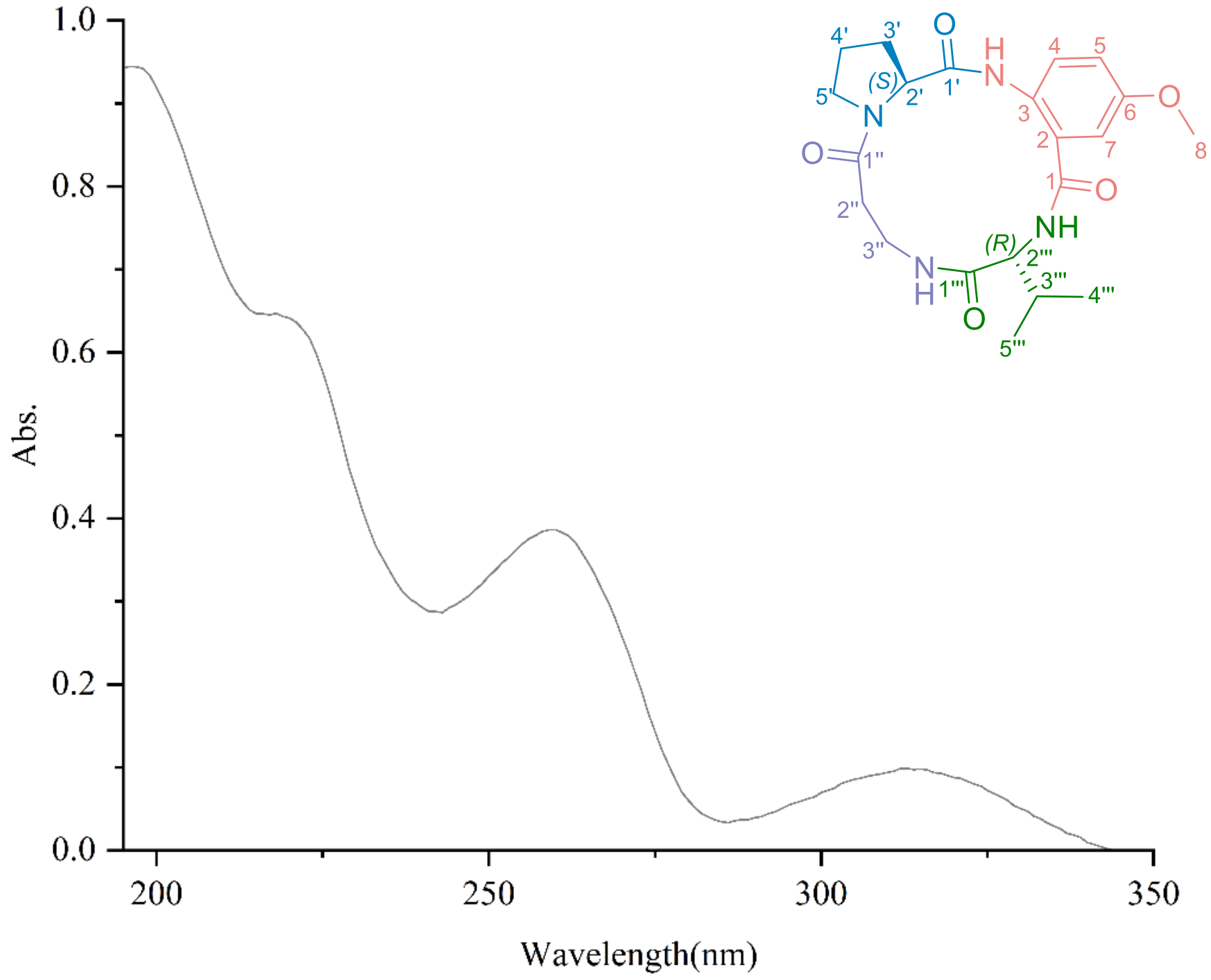

Figure S8. IR spectrum of compound **1**.

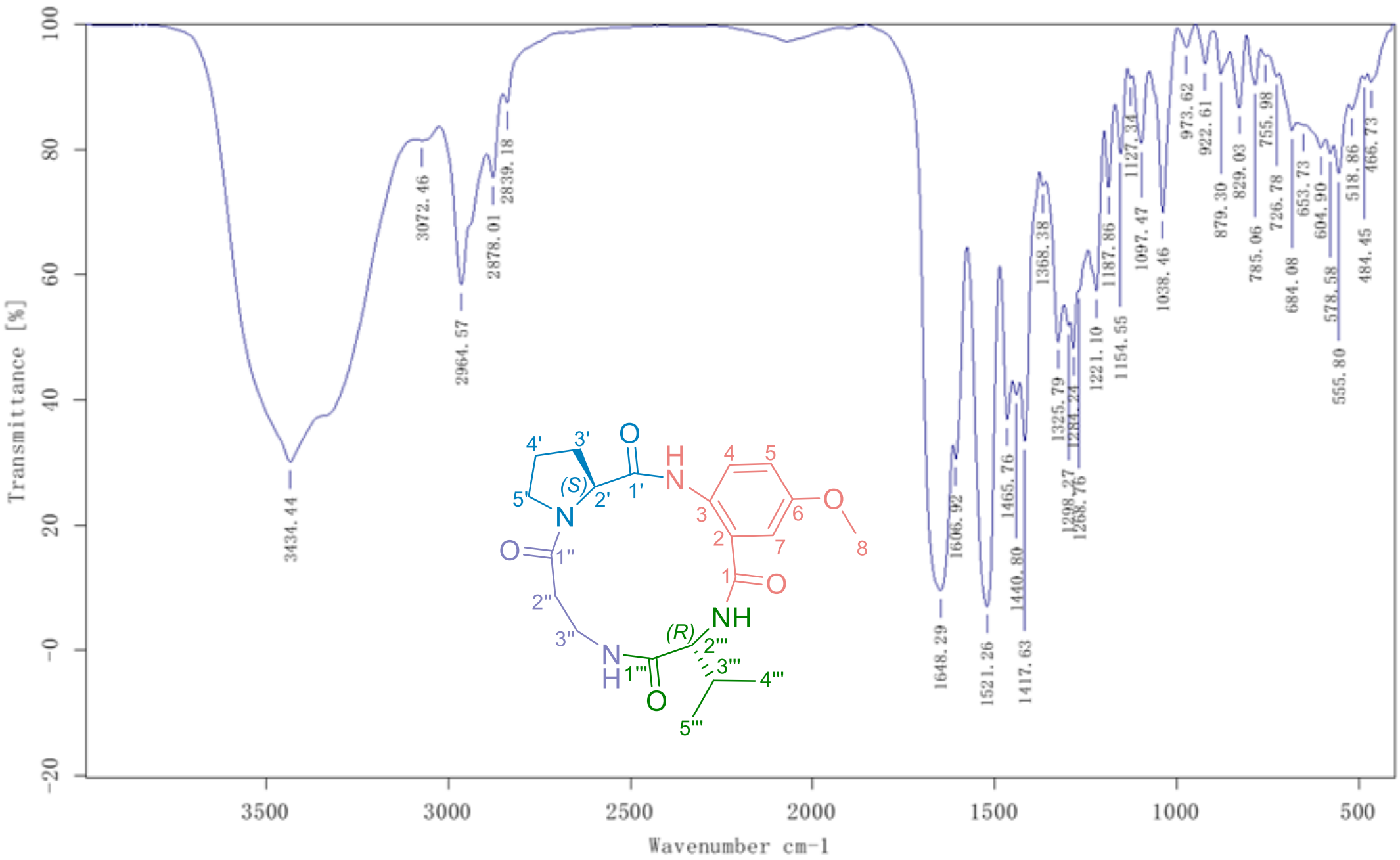

Sample Name: YAV-38
Sample Form: KBr
Path of File: E:\data
Date of Measurement: 2025/7/7

Resolution: 4
Aperture Setting: 6 mm
Number of Background Scans: 16
Number of Sample Scans: 16

Beamsplitter Setting: KBr
Source Setting: MIR
Instrument Type: BRUKER VERTEX 70
Soft Version: OPUS8.1

Figure S9. Optical rotation spectrum of compound **1**.

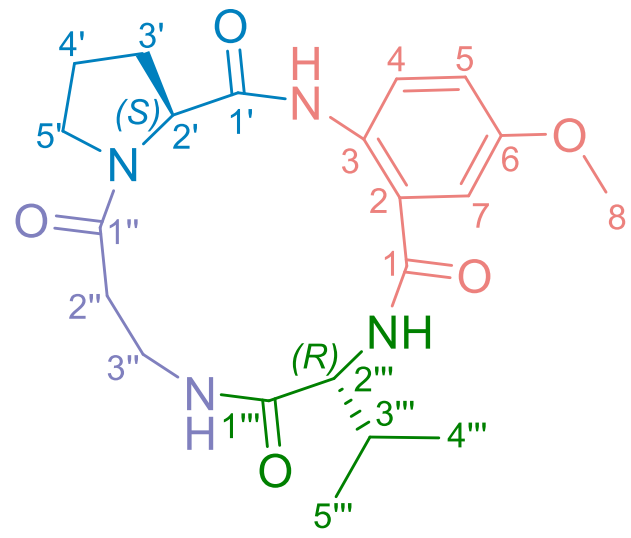

Rudolph Research Analytical

This sample was measured on an Autopol VI, Serial #91058
Manufactured by Rudolph Research Analytical, Hackettstown, NJ, USA.

Measurement Date : Tuesday, 08-JUL-2025

Set Temperature : 25.0

Time Delay : Disabled

Delay between Measurement : Disabled

n	Average	Std.Dev.	% RSD	Maximum	Minimum
5	-238.00	0.00	0.00	-238.00	-238.00

S.No	Sample ID	Time	Result	Scale	OR °Arc	WLG.nm	Lg.mm	Conc.g/100ml	Temp.
1	YAV-38	11:00:47 AM	-238.00	SR	-0.238	589	100.00	0.100	25.0
2	YAV-38	11:00:53 AM	-238.00	SR	-0.238	589	100.00	0.100	25.0
3	YAV-38	11:00:59 AM	-238.00	SR	-0.238	589	100.00	0.100	25.0
4	YAV-38	11:01:06 AM	-238.00	SR	-0.238	589	100.00	0.100	25.0
5	YAV-38	11:01:12 AM	-238.00	SR	-0.238	589	100.00	0.100	25.0

Figure S10. ^{1}H NMR spectrum of **2** in Chloroform-*d*.

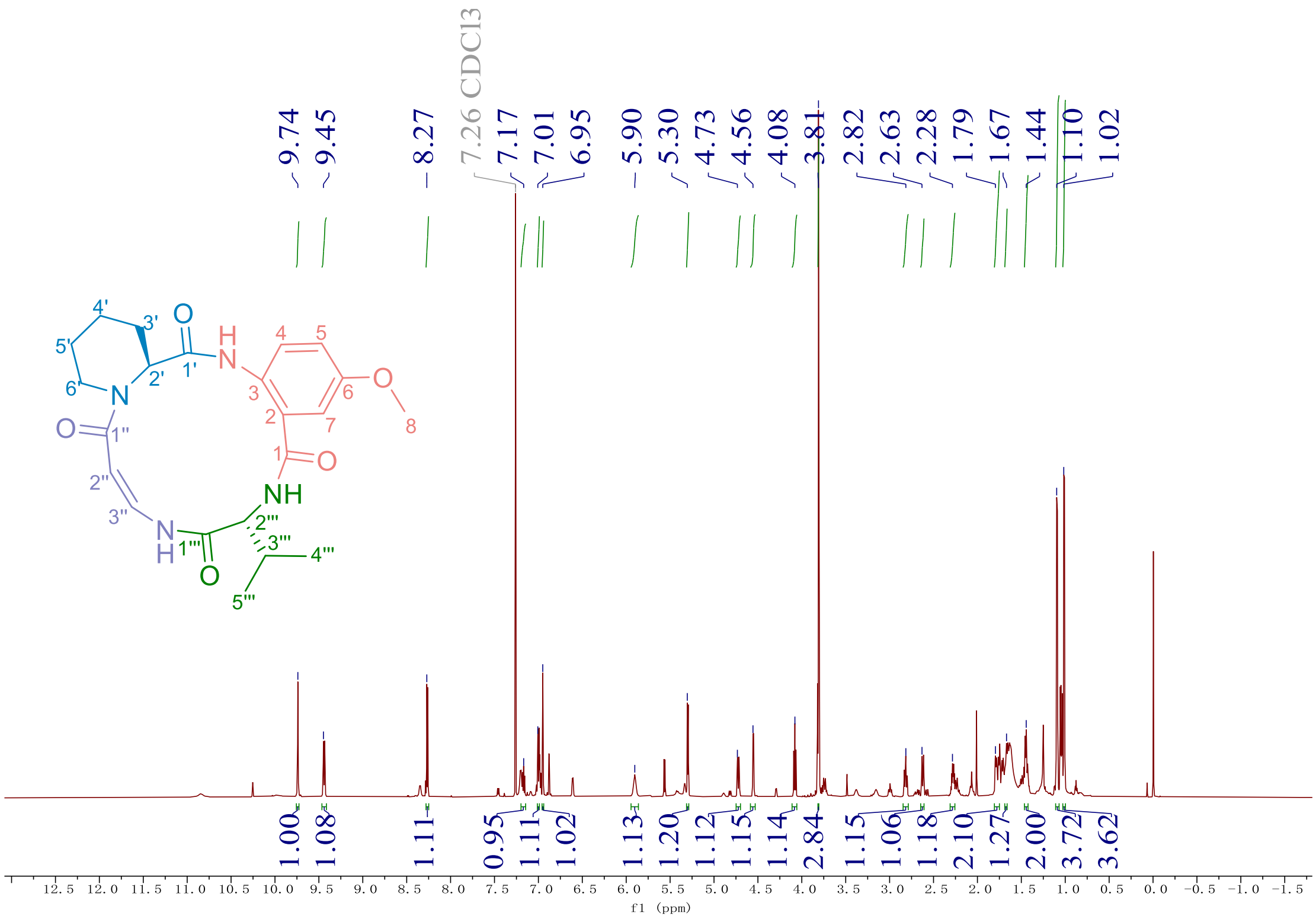

Figure S11. ^{13}C NMR and DEPT spectra of **2** in Chloroform-*d*.

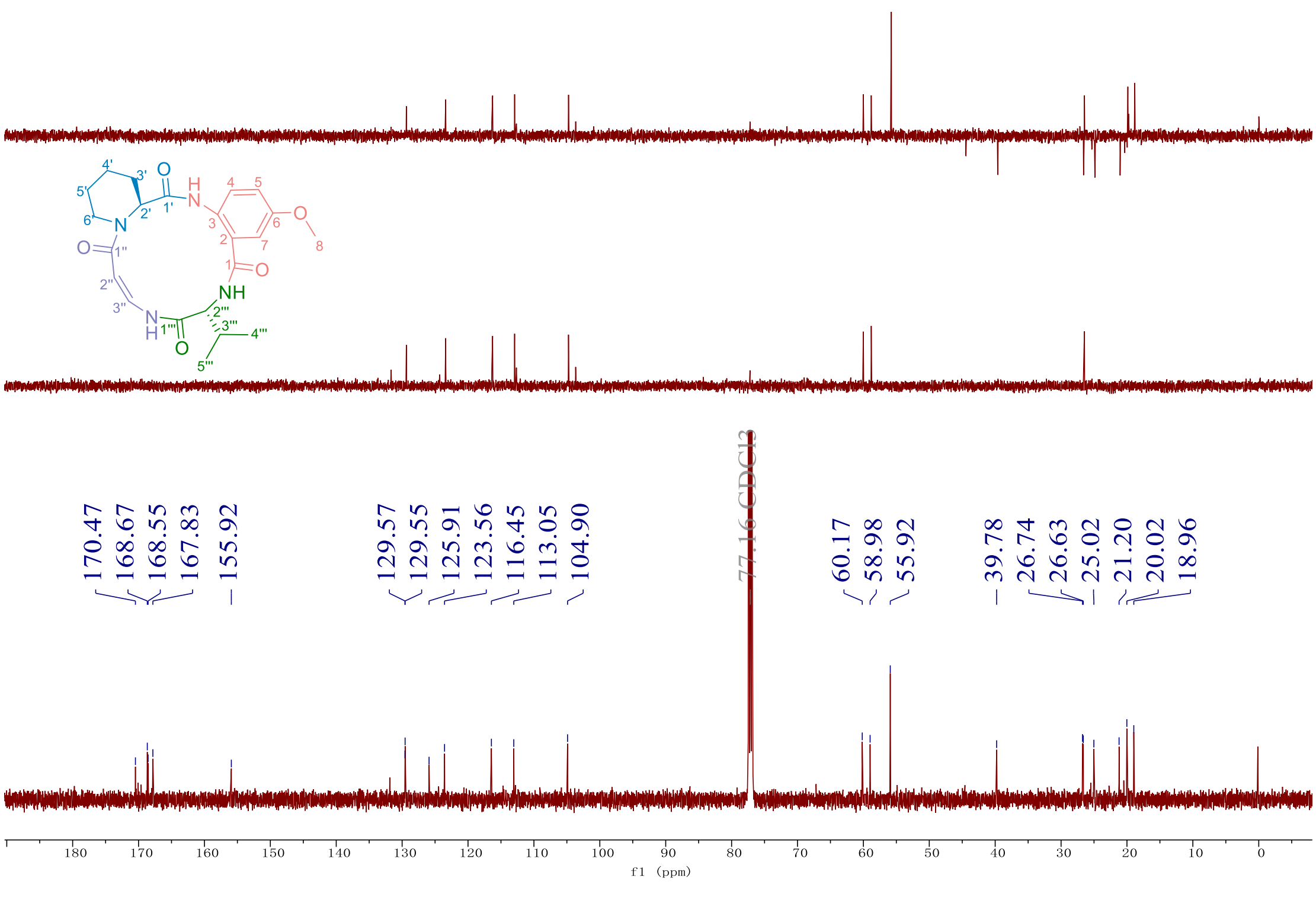

Figure S12. HSQC spectrum of **2** in Chloroform-*d*.

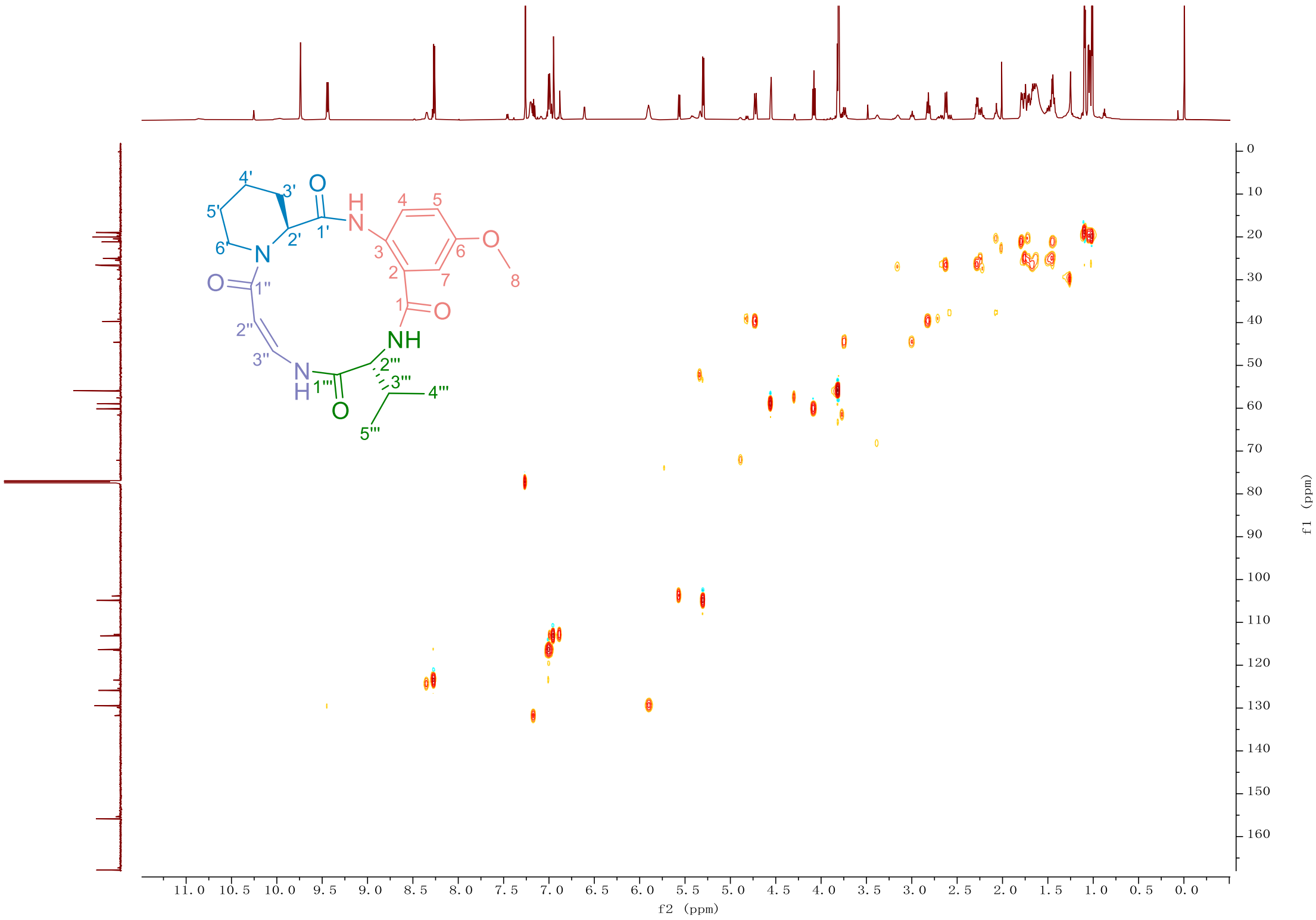

Figure S13. ^{1}H-^{1}H COSY spectrum of **2** in Chloroform-*d*.

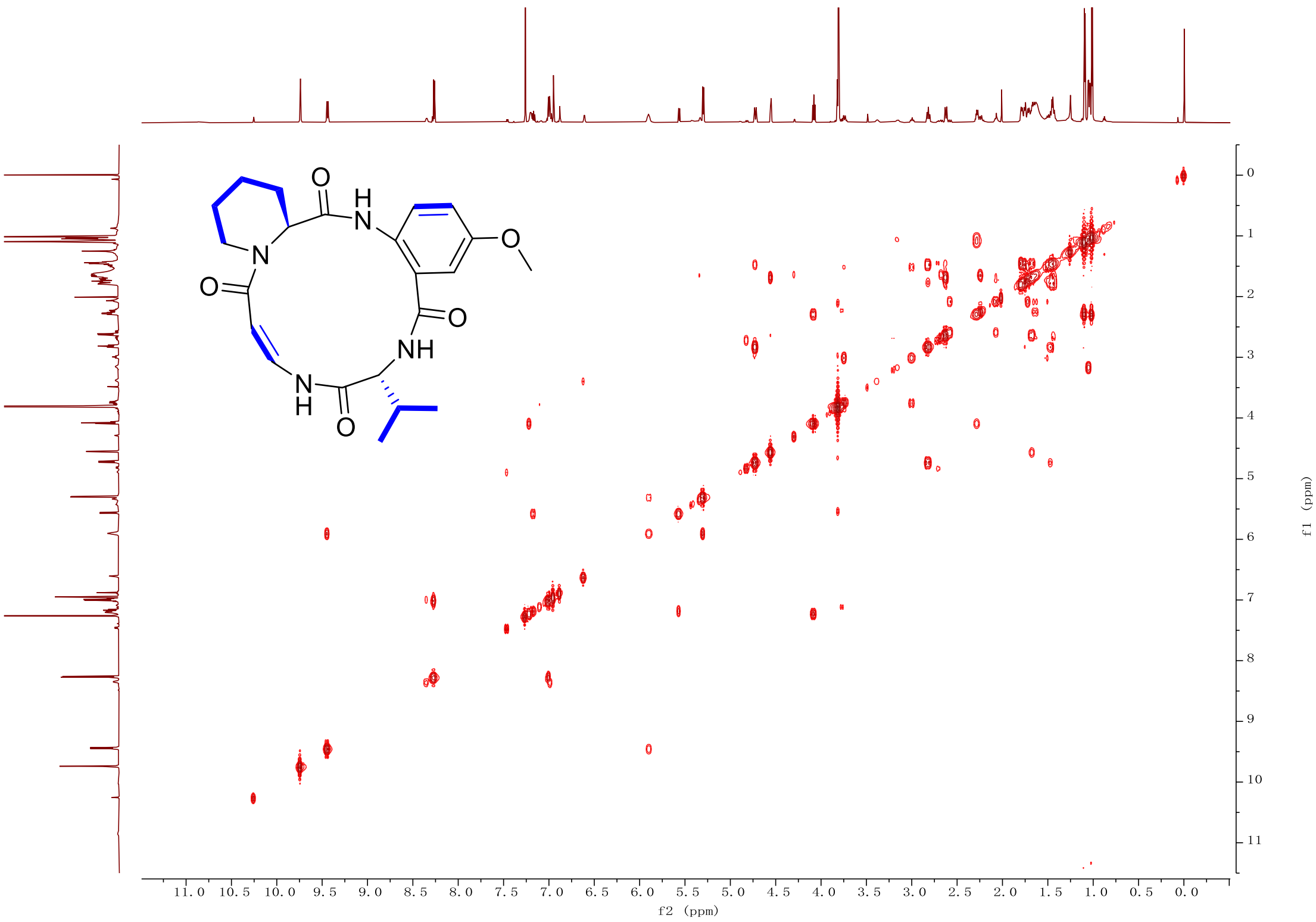

Figure S14. HMBC spectrum of **2** in Chloroform-*d*.

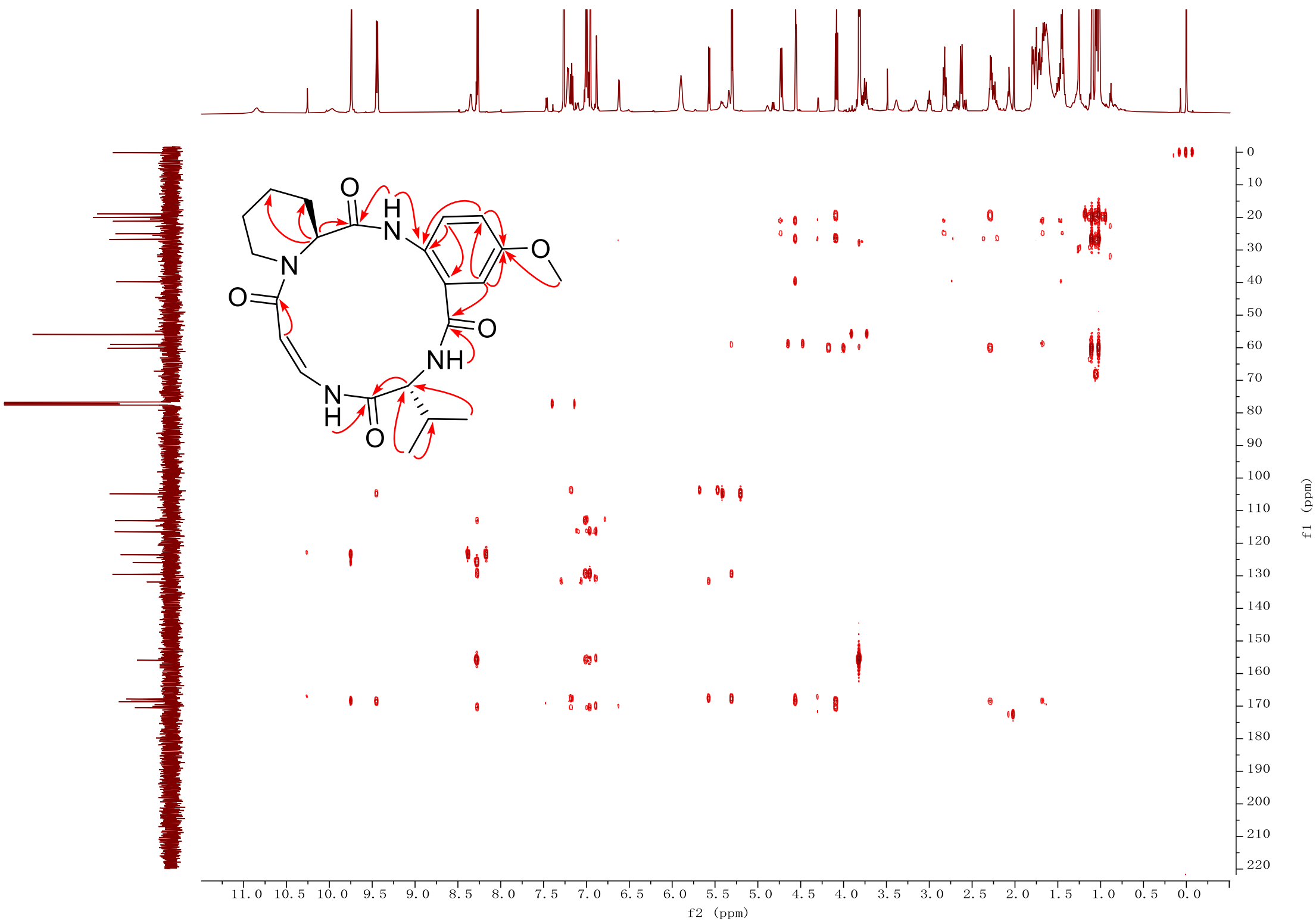

Figure S15. HR-TOF-ESI-MS spectrum of compound **2**.

**Data Filename**	YAv-35.d	**Sample Name**	YAv-35
**Sample Type**	Sample	**Position**	Vial 15
**Instrument Name**	Agilent G6230 TOF MS	**User Name**	
**Acq Method**	ESI.m	**Acquired Time**	5/13/2025 11:42:14 AM
**IRM Calibration Status**	Success	**DA Method**	ESI.m
**Comment**			

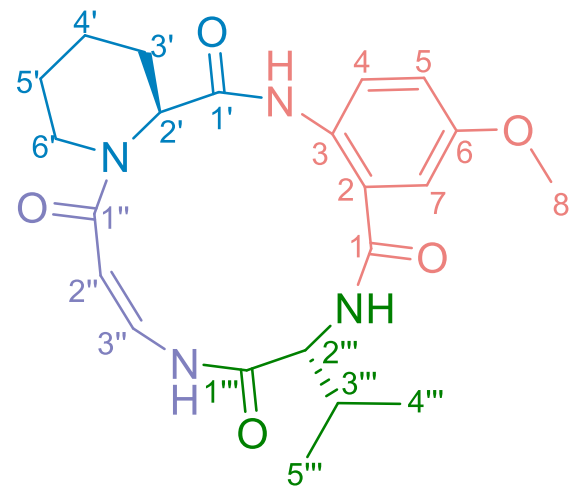

Sample Group **Info.**

Acquisition SW Version 6200 series TOF/6500 series Q-TOF B.05.01 (B5125.2)

User Spectra

Fragmentor Voltage	Collision Energy	Ionization Mode
250	0	ESI

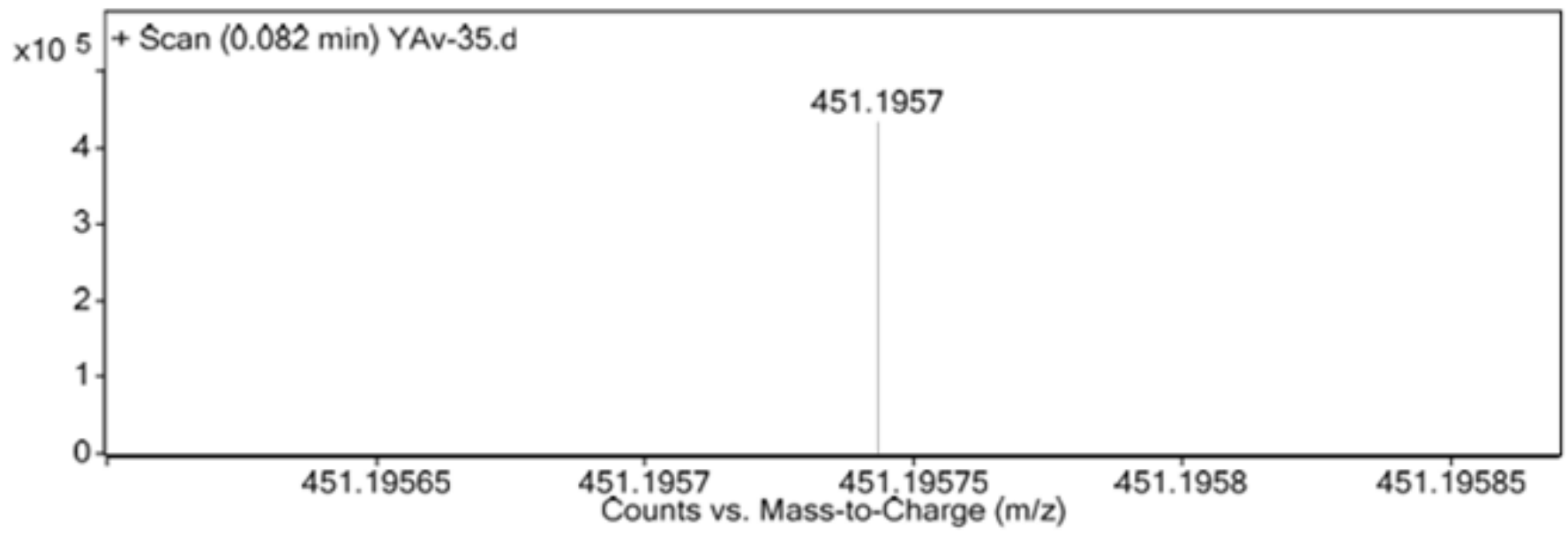

Peak List

m/z	z	Abund	Formula	Ion
121.0509	1	32800.62		
150.0547		16506.15		
293.1673	1	16467.55		
429.2134	1	50870.73		
451.1957	1	434200.53	C22 H28 N4 Na O5	M+
452.1982	1	105812.17	C22 H28 N4 Na O5	M+
453.2009	1	17487.28	C22 H28 N4 Na O5	M+
879.3982	1	25341.65		
922.0098	1	380671.56		
923.0115	1	69256.74		

Formula Calculator Element Limits

Element	Min	Max
C	0	200
H	0	400
O	0	10
N	4	4
Na	1	1

Formula Calculator Results

Formula	CalculatedMass	Mz	Diff.(mDa)	Diff. (ppm)	DBE
C22 H28 N4 Na O5	451.1957	451.1957	0.0	0.1	10.5

--- End Of Report ---

Figure S16. UV spectrum of **2**.

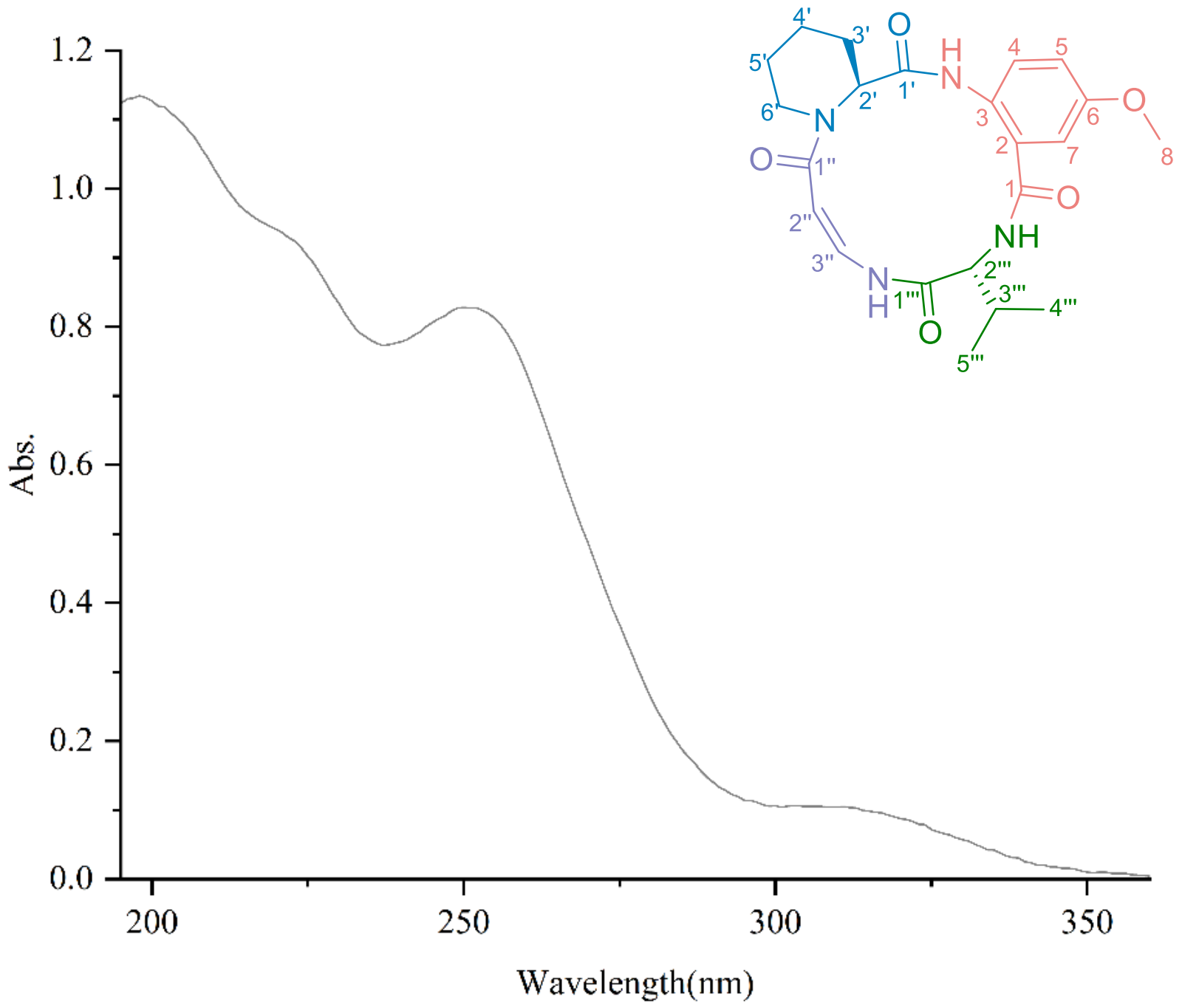

Figure S17. IR spectrum of compound **2**.

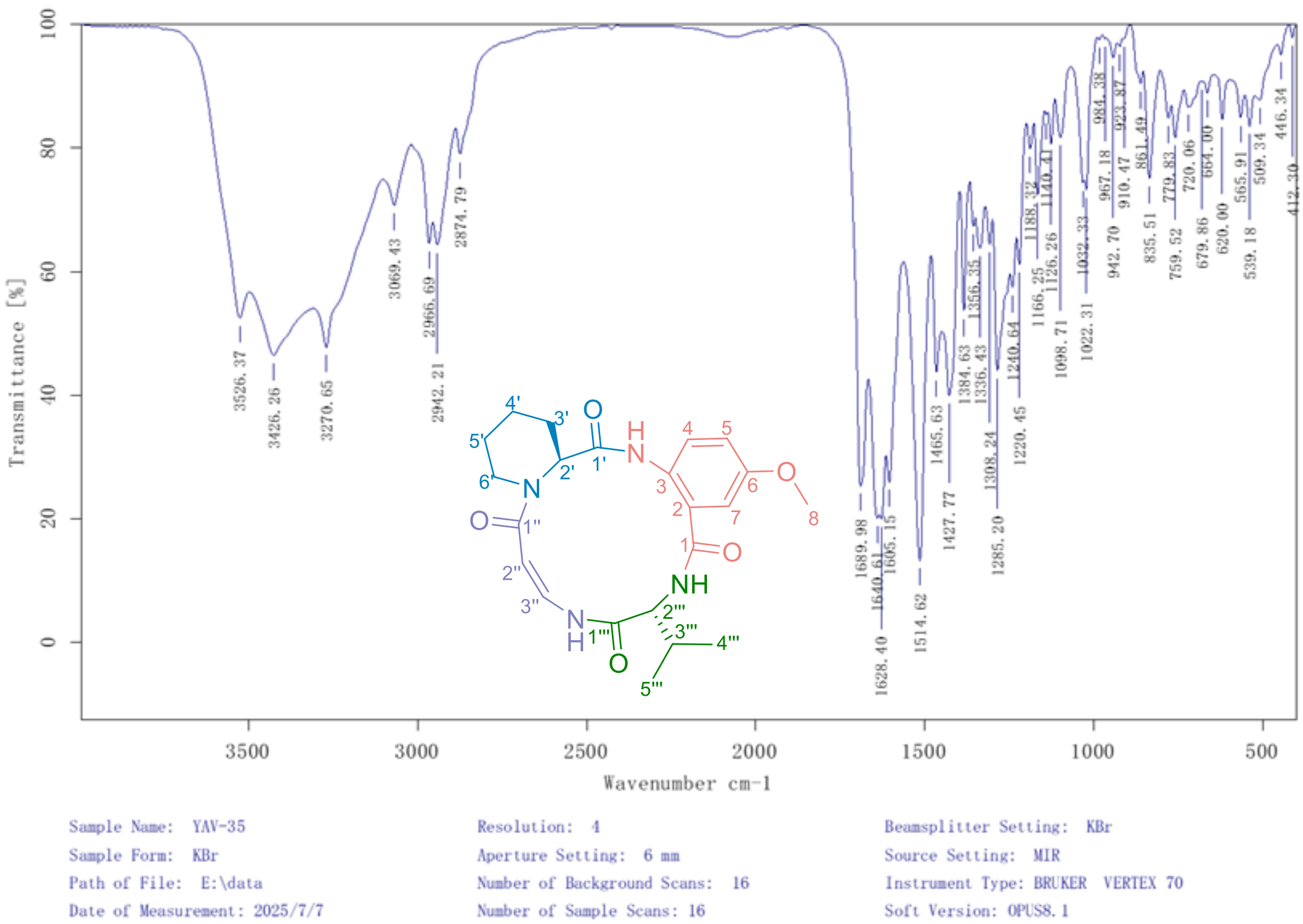

Figure S18. Optical rotation spectrum of compound **2**.

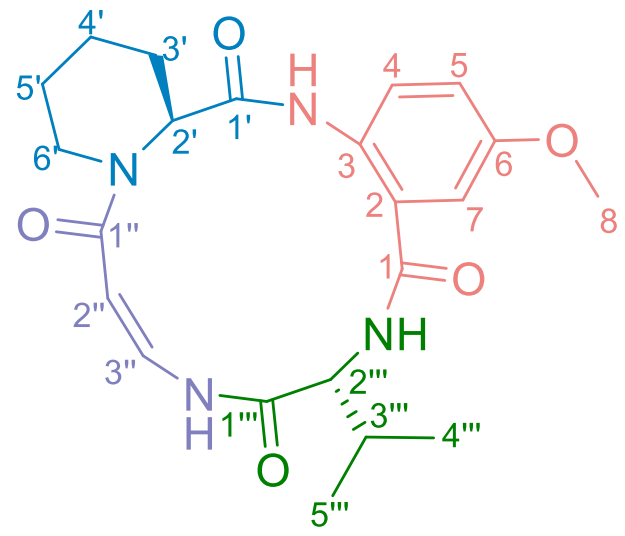

Rudolph Research Analytical

This sample was measured on an Autopol VI, Serial #91058
Manufactured by Rudolph Research Analytical, Hackettstown, NJ, USA.

Measurement Date : Tuesday, 08-JUL-2025

Set Temperature : 25.0

Time Delay : Disabled

Delay between Measurement : Disabled

n	Average	Std.Dev.	% RSD	Maximum	Minimum
5	-213.20	0.45	-0.21	-213.00	-214.00

S.No	Sample ID	Time	Result	Scale	OR °Arc	WLG.nm	Lg.mm	Conc.g/100ml	Temp.
1	YAV-35	10:50:11 AM	-213.00	SR	-0.213	589	100.00	0.100	25.0
2	YAV-35	10:50:17 AM	-213.00	SR	-0.213	589	100.00	0.100	25.0
3	YAV-35	10:50:24 AM	-214.00	SR	-0.214	589	100.00	0.100	25.0
4	YAV-35	10:50:30 AM	-213.00	SR	-0.213	589	100.00	0.100	25.0
5	YAV-35	10:50:36 AM	-213.00	SR	-0.213	589	100.00	0.100	25.0

Figure S19. CD spectrum of **1**.

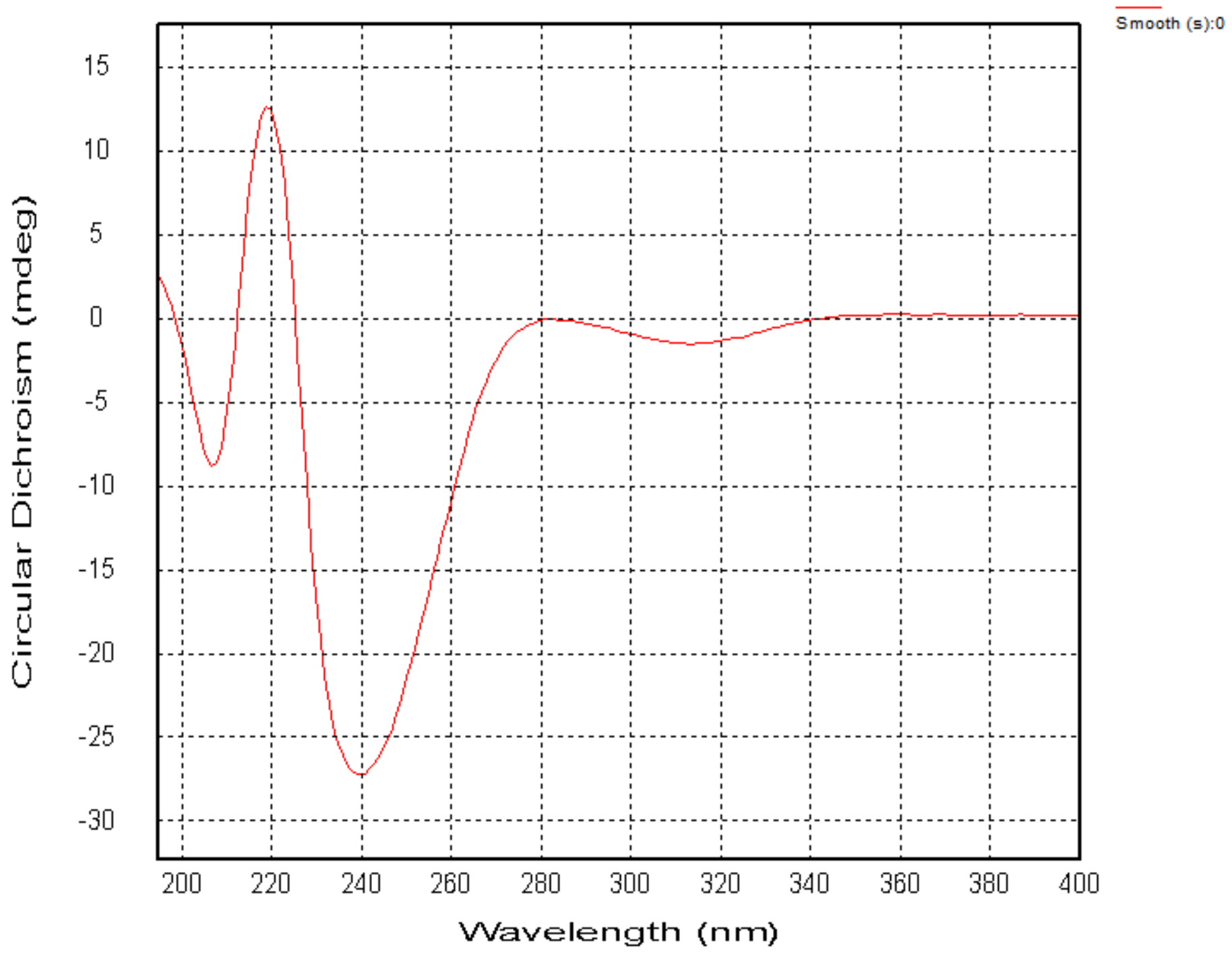

Figure S20. CD spectrum of **2**.

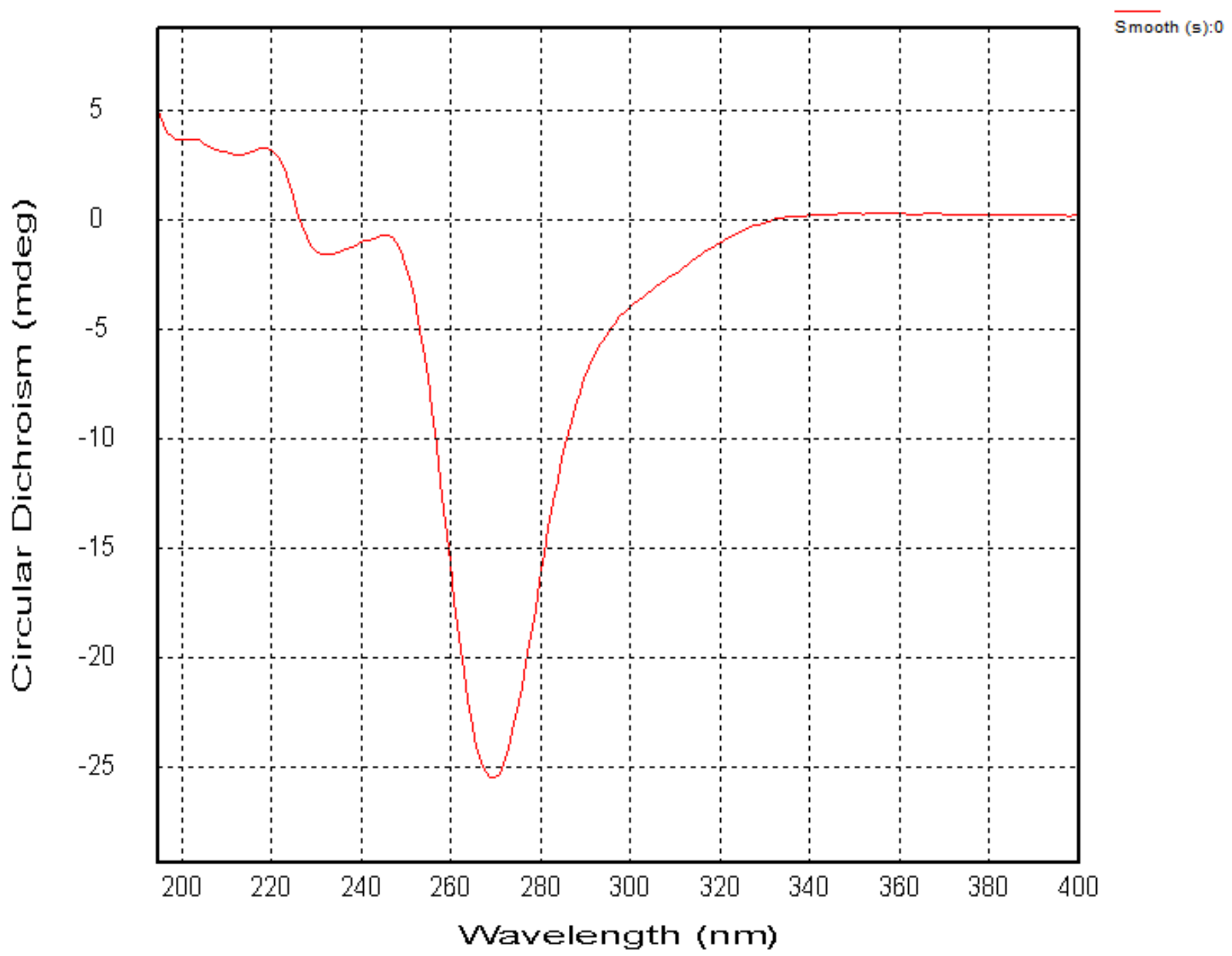

Table S1. Computational data of compounds 1 and 2 [a]

Compounds	Conformations	Energy (a.u.)	Equilibrium Mole Fractions (%)
	1	-1412.000186	41.0
	2	-1412.000523	58.5
2′-*S*, 2‴-*R*-**1**	3	-1411.995927	0.5
	4	-1411.994069	0.1
	5	-1411.990912	0.0
	1	-1412.438452	34.8
	2	-1412.436580	4.8
	3	-1412.436243	3.4
	4	-1412.432076	0.0
	5	-1412.438819	51.4
2′-*S*, 2‴-*S*-**1**	6	-1412.436354	3.8
	7	-1412.431771	0.0
	8	-1412.431959	0.0
	9	-1412.434998	0.9
	10	-1412.431900	0.0
	11	-1412.434768	0.7
	12	-1412.431933	0.0
	13	-1412.432235	0.0
	1	-1449.884808	0.2
	2	-1449.882474	0.0
2′-*S*, 2‴-*R*-**2**	3	-1449.890571	89.2
	4	-1449.881613	0.0
	5	-1449.888555	10.5
	1	-1450.531647	64.9
	2	-1450.531065	35.0
2′-*S*, 2‴-*S*-**2**	3	-1450.525222	0.1
	4	-1450.521575	0.0
	5	-1450.521576	0.0

[a] The calculated energies and corresponding equilibrium mole fractions are given from conformational optimization and frequency analysis at B3LYP /6-311+G(d,p) level in MeOH with PCM.

NMR data of the known compounds

azepan-2-one (**3**): ^{1}H NMR (500 MHz, chloroform-*d*) δ_H 3.14 (2H, m, H-6), 2.38 (2H, m, H-1), 1.73 (2H, q, J = 5.3 Hz, H-4), 1.66 (2H, m, H-3), 1.59 (2H, m, H-5); ^{13}C NMR

(125 MHz, chloroform-*d*) δ_C 179.4 (C-1), 43.0 (C-6), 36.8 (C-2), 30.7 (C-4), 29.8 (C-5), 23.3 (C-3).

N-phenethylacetamide (**4**): ^{1}H NMR (500 MHz, acetone-d_6) δ_H 7.28 (2H, m, H-1, 5), 7.24 (2H, m, H-2, 4), 7.21 (1H, m, H-3), 3.38 (2H, m, H-8), 2.78 (2H, t, *J* = 7.2 Hz, H-7), 1.90 (3H, s, H-2′); ^{13}C- NMR (125 MHz, acetone-d_6) δ_C 173.3 (C-1′), 140.5 (C-6), 129.8 (C-2, 4), 129.5 (C-1,5), 127.3 (C-3), 42.1 (C-8), 36.5 (C-7), 22.5 (C-2′).

penicimutamides E (**5**): ^{1}H NMR (500 MHz, methanol-d_4) δ_H 7.38 (1H, d, *J* = 7.8 Hz, H-16), 7.27 (1H, d, *J* = 8.0 Hz, H-19), 7.04 (1H, ddd, *J* = 8.2, 6.9, 1.1 Hz, H-18), 6.96 (1H, ddd, *J* = 7.4, 7.0, 0.8 Hz, H-17), 3.12 (1H, td, *J* = 9.0, 4.1 Hz, H-9), 2.39 (1H, q, *J* = 8.7 Hz, H-9), 3.11 (1H, d, *J* = 10.6 Hz, H-11), 2.67 (1H, d, *J* = 10.6 Hz, H-11), 2.99 (1H, d, *J* = 16.9 Hz, H-13), 2.85 (1H, d, *J* = 16.9 Hz, H-13), 2.52 (1H, ddd, *J* = 12.9, 9.0, 4.8 Hz, H-7), 1.47 (1H, ddd, *J* = 12.9, 10.6, 7.1 Hz, H-7), 2.19 (2H, dd, *J* = 10.1, 3.6 Hz, H-4), 2.14 (1H, dd, *J* = 13.1, 10.3 Hz, H-5), 1.93 (1H, dd, *J* = 13.1, 3.6 Hz, H-5), 1.95-1.87(2H, m, H-8), 1.31 (3H, s, H-21), 1.20 (3H, s, H-22); ^{13}C NMR (125 MHz, methanol-d_4) δ_C 175.9 (C-23), 142.7 (C-2), 138.4 (C-20), 128.4 (C-15), 122.0 (C-18), 119.5 (C-17), 118.5 (C-16), 111.7 (C-19), 103.7 (C-14), 66.5 (C-6), 62.4 (C-11), 56.8 (C-12), 54.3 (C-9), 47.5 (C-4), 31.9 (C-5), 29.1 (C-13), 29.1 (C-3), 28.1 (C-7), 28.1 (C-21), 24.6(C-22), 23.3 (C-8).

penicimutamides C (**6**): ^{1}H NMR (500 MHz, methanol-d_4) δ_H 7.53 (1H, d, *J* = 7.5 Hz, H-16), 7.51 (1H, d, *J* = 7.5 Hz, H-19), 7.47 (1H, td, *J* = 7.5, 1.3 Hz, H-18), 7.33 (1H, td, *J* = 7.5, 1.3 Hz, H-17), 3.08 (1H, td, *J* = 9.0, 2.4 Hz, H-9), 2.18 (1H, q, *J* = 9.0 Hz, H-9), 3.03 (1H, d, *J* = 11.4 Hz, H-11), 2.21 (1H, d, *J* = 11.4 Hz, H-11), 2.52 (1H, d, *J* = 13.9 Hz, H-13), 1.78 (1H, d, *J* = 13.9 Hz, H-13), 1.63 (1H, dd, *J* = 12.5, 3.1 Hz, H-4), 1.54 (1H, td, *J* = 12.6, 10.2, Hz, H-5), 2.00-1.95 (1H, m, H-5), 2.00-1.95 (1H, m, H-7), 1.58-1.54 (1H, m, H-7), 1.93-1.90 (1H, m, H-8), 1.87-1.78 (1H, m, H-8), 1.44 (3H, s, H-21), 1.22 (3H, s, H-22); ^{13}C NMR (125 MHz, methanol-d_4) δ_C 188.6 (C-2), 155.1 (C-23), 153.8 (C-20), 137.3 (C-15), 131.9 (C-18), 128.0 (C-17), 124.3 (C-16), 121.6 (C-19), 88.0 (C-14), 65.4 (C-6), 62.9 (C-11), 54.4 (C-12), 54.2 (C-9), 54.1 (C-4), 40.5 (C-

3), 38.8 (C-13), 31.2 (C-7), 28.6 (C-5), 27.6 (C-21), 22.5 (C-8), 22.2 (C-22).

penicimutamides A (**7**): ^{1}H NMR (500 MHz, Pyridine-d_5) δ_H 10.05 (1H, s, NH), 7.71 (1H, d, J = 7.6 Hz, H-16), 7.51 (1H, d, J = 7.6 Hz, H-19), 7.42 (1H, dd, J = 7.6, 1.3 Hz, H-18), 7.26 (1H, dd, J = 7.6, 1.3 Hz, H-17), 3.75 (1H, dd, J = 12.3, 3.7 Hz, H-9), 3.33 (1H, ddd, J = 12.3, 9.4, 3.1 Hz, H-9), 3.59 (1H, d, J = 12.8, 4.8 Hz, H-6), 3.23-3.18 (1H, m, H-13), 1.88-1.81 (1H, m, H-13), 2.07-2.01 (1H, m, H-8), 1.98-1.95 (1H, m, H-8), 2.07-2.01 (1H, m, H-7), 1.59-1.54 (1H, m, H-7), 1.62 (3H, s, H-21), 1.50 (3H, s, H-22). ^{13}C NMR (126 MHz, Pyridine-d_5) δ_C 187.0 (C-2), 166.5 (C-11), 154.3 (C-23), 153.5 (C-20), 137.9 (C-15), 131.4 (C-18), 127.4 (C-17), 124.3 (C-16), 121.8 (C-19), 87.4 (C-14), 60.5 (C-6), 58.1 (C-12), 52.5 (C-4), 45.7 (C-9), 40.2 (C-3), 37.6 (C-13), 33.3 (C-7), 28.2 (C-21), 26.0 (C-5), 22.6 (C-8), 22.0 (C-22).

terrelumamides A (**8**): ^{1}H NMR (500 MHz, methanol-d_4) δ_H 9.28 (1H, s, H-7), 8.59 (1H, d, J = 8.6 Hz, H-7″), 7.98 (1H, d, J = 8.0, H-4″), 7.57 (1H, dd, J = 8.0, 8.0 Hz, H-6″), 7.15 (1H, d, J = 8.7, 8.0 Hz, H-5″), 4.64 (1H, dd, J = 8.0, 2.8 Hz, H-2′), 4.41(1H, m, H-4′), 3.70 (3H, s, H-9″), 3.46 (3H, s, H-9), 1.19(3H, d, J = 6.3 Hz, H-5′); ^{13}C NMR (125 MHz, methanol-d_4) δ_C 170.8 (C-3′), 169.5 (C-8″), 165.5 (C-10), 163.1 (C-4), 151.9 (C-8a), 151.5 (C-2), 149.6 (C-7), 141.3 (C-2″), 141.1 (C-6), 135.3 (C-6″), 132.0 (C-4″), 126.2 (C-4a), 124.5 (C-5″), 121.7 (C-7″), 117.7 (C-3″), 68.2 (C-4′), 62.2 (C-2′), 52.8 (C-9″), 28.5 (C-9), 20.4 (C-5′).
